# Exfoliated near infrared fluorescent silicate nanosheets for (bio)photonics

**DOI:** 10.1038/s41467-020-15299-5

**Published:** 2020-03-20

**Authors:** Gabriele Selvaggio, Alexey Chizhik, Robert Nißler, llyas Kuhlemann, Daniel Meyer, Loan Vuong, Helen Preiß, Niklas Herrmann, Florian A. Mann, Zhiyi Lv, Tabea A. Oswald, Alexander Spreinat, Luise Erpenbeck, Jörg Großhans, Volker Karius, Andreas Janshoff, Juan Pablo Giraldo, Sebastian Kruss

**Affiliations:** 1 Institute of Physical Chemistry, University of Göttingen, Göttingen, 37077 Germany; 2 Third Institute of Physics, University of Göttingen, Göttingen, 37077 Germany; 3 Institute of Organic and Biomolecular Chemistry, University of Göttingen, Göttingen, 37077 Germany; 4 Institute of Developmental Biochemistry, Medical School, University of Göttingen, Göttingen, 37077 Germany; 5 Department of Dermatology, Venereology and Allergology, University Medical Center Göttingen, Göttingen, 37075 Germany; 6 Department of Sedimentology and Environmental Geology, Geoscience Center, University of Göttingen, Göttingen, 37077 Germany; 7 Department of Botany and Plant Sciences, University of California, Riverside, California 92507 USA

**Keywords:** Fluorescence imaging, Nanoscale biophysics, Nanophotonics and plasmonics, Imaging techniques, Nanoparticles

## Abstract

Imaging of complex (biological) samples in the near-infrared (NIR) is beneficial due to reduced light scattering, absorption, phototoxicity, and autofluorescence. However, there are few NIR fluorescent materials known and suitable for biomedical applications. Here we exfoliate the layered pigment CaCuSi_4_O_10_ (Egyptian Blue, EB) via ball milling and facile tip sonication into NIR fluorescent nanosheets (EB-NS). The size of EB-NS can be tailored to diameters <20 nm and heights down to 1 nm. EB-NS fluoresce at 910 nm and the fluorescence intensity correlates with the number of Cu^2+^ ions. Furthermore, EB-NS display no bleaching and high brightness compared with other NIR fluorophores. The versatility of EB-NS is demonstrated by in-vivo single-particle tracking and microrheology measurements in *Drosophila melanogaster* embryos. EB-NS can be uptaken by plants and remotely detected in a low-cost stand-off detection setup. In summary, EB-NS have the potential for a wide range of bioimaging applications.

## Abstract (web summary)

Near-infrared (NIR) fluorophores have attracted interest for bioimaging; yet availability, biocompatibility and application can be an issue. Here, the authors report on the development of Egyptian Blue nanosheets with high NIR fluorescence and photostability demonstrating bioimaging applications in vivo.

## Introduction

Fluorescence imaging provides important insights into the structure, function, and dynamics of biological samples^1,2^. Imaging in the near-infrared (NIR) spectral range (800–1700 nm) promises higher tissue penetration, higher contrast, and lower phototoxicity due to reduced NIR light scattering and absorption^3–5^. However, these approaches are limited by the scarcity of NIR fluorescent materials. NIR fluorescent organic dyes such as indocyanine green (ICG) bleach and are therefore not suitable for long-term imaging^6,7^. In contrast, nanomaterials provide beneficial photophysical properties such as ultra-high photostability that would enable tracking in living systems without time constrains. NIR fluorescent nanomaterials include InAs quantum dots, lanthanide-doped nanoparticles, or semiconducting single-walled carbon nanotubes (SWCNTs)^8–12^. For example, SWCNTs have been used as building blocks for NIR imaging and as fluorescent sensors that detect small signaling molecules, proteins, or lipids^13–19^. They can be chemically tailored and have been used to reveal spatiotemporal release patterns of neurotransmitters from single cells^2,15,20–22^. However, most NIR fluorescent nanomaterials often have low quantum yields, lack biocompatibility, or are restricted to certain emission/excitation wavelengths. Therefore, there is a major need for novel NIR fluorescent and biocompatible nanomaterials for sophisticated applications such as long-time single-particle tracking in organisms or multiscale bioimaging such as stand-off detection in plants^23,24^.

One of the first colored pigments created by mankind is the calcium copper silicate called Egyptian Blue (CaCuSi_4_O_10_, EB), which has been synthesized and used as early as 2500 BC in Ancient Egypt^25^. Current ancient works of art decorated with EB have lost none of their vibrant color, a testimony to the remarkable chemical stability of this compound. Interestingly, bulk EB displays NIR fluorescence, which was only recently identified^26,27^ and attributed to a ^2^B_2g_-^2^B_1g_ electronic transition of the copper ion that ranges from 910 to 930 nm^26,28^. Bulk EB shows a remarkable high quantum yield of 10.5% for a NIR emitter compared with SWCNTs, quantum dots, metal nanoclusters, and FDA-approved fluorophores such as ICG^5,28^. Recently, micrometer-sized monolayer sheets of EB were isolated by stirring in hot water for several days^29,30^. However, the remarkable properties of EB have not been explored for developing NIR luminescent nanomaterials for bioimaging applications. The layered structure of EB suggests that exfoliation procedures known from other two-dimensional (2D) materials including graphene and transition metal chalcogenides could efficiently exfoliate it^31–34^. Such 2D materials have been shown to possess promising optoelectronic properties and are a rich playground for physics and chemistry^35^.

Herein, we use a facile tip sonication technique to exfoliate CaCuSi_4_O_10_ (EB) nanosheets (EB-NS). This procedure allows one to control the nanomaterial size/thickness and retain the unique NIR fluorescent properties of macroscopic CaCuSi_4_O_10_. We report the photophysical properties of EB-NS and investigate how NIR fluorescence scales with nanosheet size. Furthermore, we demonstrate the use of this material for in-vivo NIR microscopy and stand-off detection.

## Results

### Exfoliation of Egyptian Blue into nanosheets

**Fig. 1:**
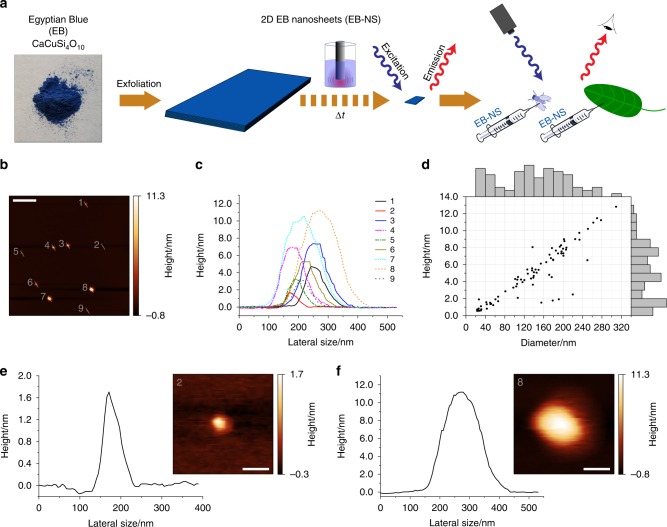
Exfoliation of Egyptian Blue (EB) into Egyptian Blue nanosheets (EB-NS). **a** EB bulk powder (CaCuSi_4_O_10_, photograph taken in our lab) is exfoliated into EB-NS via tip sonication to study their near-infrared (NIR) photophysical properties. These nanostructures are furthermore used for in-vivo NIR imaging in plants and *Drosophila* embryos. **b** Representative AFM image of EB-NS. Scale bar = 1 µm. **c** Corresponding height profiles (highlighted by a white line in **b**). **d** Height and diameter distribution after 6 h of tip sonication. The bars at the axis are histograms. *n* = 78 EB-NS. **e**, **f** Height profiles and magnified images of the smallest and largest nanosheets shown in **b**. Scale bar = 100 nm.

The size of a nanomaterial determines its properties and interactions with the environment. For fluorescence imaging in cells or whole organisms, fluorophores should be as small as possible to not perturb the system. Exfoliation into 2D sheets is one step but it is also important to decrease the lateral size into the nanoscale. Therefore, we first reduced the size of EB by planetary ball (PB) milling and then exfoliated EB-NS via tip sonication, which allowed for the controlled decrease in height and diameter with sonication time (Fig. 1a and Supplementary Fig. 1).

Milling techniques such as PB are routinely employed for silicates^36^, whereas tip sonication is a widely used method to disperse nanomaterials such as carbon nanotubes^37^. Tip sonication for 6 h in isopropanol yielded nanosheets of lateral sizes between 20 and 300 nm, and heights of around 1–13 nm (Fig. 1b–d). Atomic force microscopy (AFM) images (Fig. 1b, c) indicate that EB height correlated linearly with diameter (Fig. 1d). The height profiles of EB-NS (Fig. 1e, f) typically contain a peak, which can be explained by the convolution through the AFM tip. The smallest observed height (≈1 nm) corresponds to the monolayer of EB bulk material reported in literature^29,38,39^ (Supplementary Fig. 2), but the exact height measured with AFM might vary depending on hydration and underlying substrate morphology^29^. To further purify EB-NS samples, we typically used syringe cut-off filters (0.20 µm/0.45 µm) to remove the remaining larger particles from the samples.

### Photophysical properties of EB-NS

**Fig. 2:**
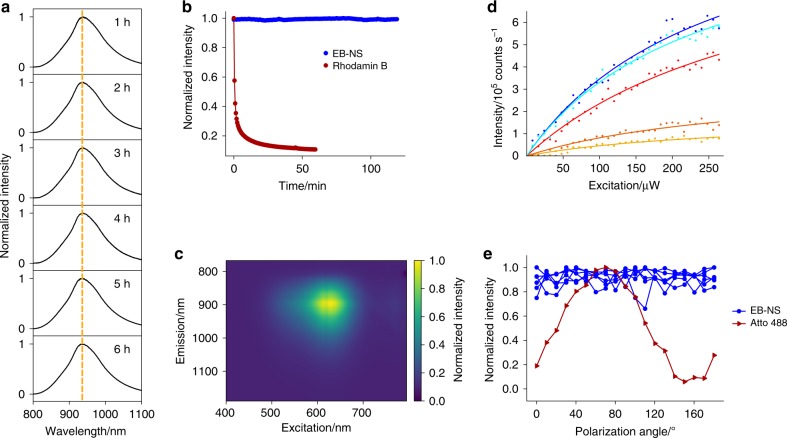
NIR fluorescence properties of EB-NS. **a** Normalized NIR fluorescence spectra of EB-NS after different tip sonication times show no wavelength shifts. **b** EB-NS fluorescence (NIR) under continuous laser excitation (561 nm, 100 mW, corresponding to about 1 W cm^−^^2^) does not bleach. In contrast, the fluorescent dye Rhodamin B (in the visible) quickly bleaches. **c** 2D excitation-emission spectrum of EB-NS. **d** Fluorescence saturation curves of different EB-NS (size below the resolution limit). **e** EB-NS display no fluorescence polarization. The dye Atto 488 is used as reference due to its distinct fluorescence polarization.

In a next step, we explored how reducing the size and dimensionality of EB into the nanoscale regime affects its luminescence properties relative to macroscopic EB powder^27^. Fluorescence quantum yields of one-dimensional materials such as SWCNTs have been shown to decrease with size, probably due to exciton diffusion and their collision with SWCNT ends^40^. Interestingly, EB fluorescence spectra did not change/shift with longer sonication times (Fig. 2a) corresponding to smaller dimensions (Fig. 1). EB-NS are extremely photostable as evidenced by the constant fluorescence emission intensity over several hours compared with the rapid bleaching of a typical organic dye (Fig. 2b). These measurements were performed on an organic dye (Rhodamin B) and on EB-NS, which were adsorbed on a glass surface under continuous excitation with a 561 nm laser (100 mW total power output) for >2 h. In addition, EB-NS show no change or shift in fluorescence emission in the presence of small redox active molecules that are known to affect the fluorescence of many dyes and fluorescent nanomaterials (Supplementary Fig. 3). EB-NS fluorescence is characterized by a large Stokes shift with a single emission maximum at ≈910 nm and an absorption maximum at ≈630 nm, as shown in the 2D excitation-emission spectrum and in the reflectance spectrum (Fig. 2c and Supplementary Fig. 4). EB-NS have a similar zeta potential (−22 mV) to spherical silica nanoparticles, which highlights that they can be dispersed and applied in aqueous solutions (Supplementary Fig. 5)^41^.

**Fig. 3:**
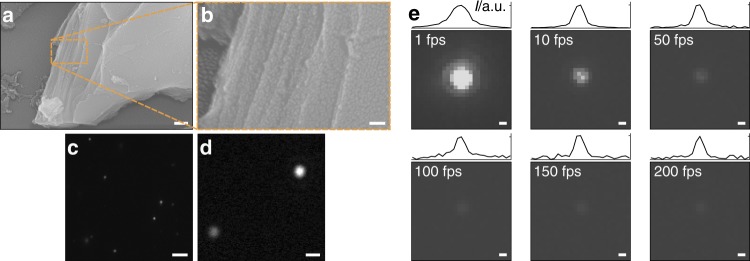
SEM and NIR imaging of EB-NS. **a**, **b** Scanning electron microscopy (SEM) images of a larger EB particle. The blow-up clearly shows the typical lamellar structure of this material. Scale bar = 200 nm and 50 nm (respectively). **c**, **d** NIR images of EB-NS taken with a Si-based camera at increasing magnification show that single EB-NS can be resolved via NIR fluorescence microscopy down to the resolution limit of light microscopy. Excitation wavelength = 561 nm. Scale bar = 5 µm and 1 µm (respectively). **e** NIR images of a single EB-NS with a diameter below the resolution limit (≈500 nm) are captured with an InGaAs camera, optimized for NIR imaging at different frame rates up to 200 fps. The upper traces show the intensity profile of the image normalized to the maximum value. Excitation wavelength = 561 nm. Scale bar = 500 nm.

**Fig. 4:**
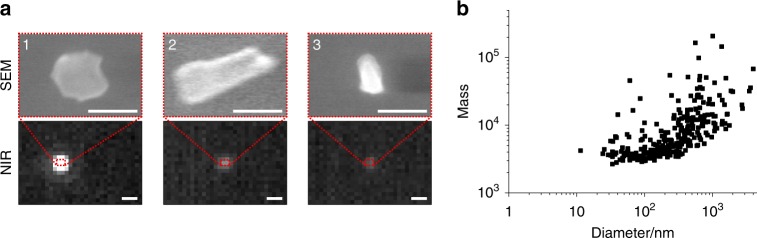
Correlative measurements of size and fluorescence intensity of EB-NS. **a** SEM and corresponding NIR images of resolution-limited EB-NS. Scale bar = 200 nm and 1 µm (respectively). **b** Fluorescence intensity as a function of hydrodynamic diameter of EB-NS obtained by the corrected Stokes–Einstein equation. Mass stands for the total integrated brightness of the particle, and the maximum value along each trajectory was taken. From this analysis, one notices that even the smallest EB-NS (≈10 nm) still fluoresce. The general trend indicates that EB-NS of larger size are brighter. *n* = 292 EB-NS.

To estimate the number of luminescent centers in a single EB-NS, we performed single-particle fluorescence saturation measurements of EB-NS using scanning confocal microscopy and pulsed laser excitation (20 MHz pulse rate). Only EB-NS below the resolution limit were studied (Fig. 2d, Supplementary Fig. 6, and Supplementary Table 1). Larger single sheets showed higher fluorescence intensities and could be distinguished from resolution-limited ones (see also Figs. 3 and 4). This finding complies with the hypothesis that the number of luminescent centers changes with the size of EB-NS. To better understand how fluorescence scales with excitation intensity, the laser power was stepwise increased and decreased, while fluorescence intensity was recorded. It was not possible to reach the emission saturation plateau with the maximum laser intensity. However, the nonlinear dependence between fluorescence intensity and laser power allowed us to fit the data with a fluorescence brightness saturation function (*I*):

1 $$I = I_{{\mathrm{sat}}}\frac{{P_{{\mathrm{exc}}}}}{{P_{{\mathrm{exc}}} + P_{{\mathrm{sat}}}}},$$

where *P*_exc_ is the excitation power, *P*_sat_ is the saturation power, and *I*_sat_ is the nanoparticle luminescence intensity that can be detected at the saturation excitation power^42^.

The obtained saturation values correspond to a fluorescence emission of 122 × 10^3^ to 1250 × 10^3^ photons s^−1^. By taking into account factors such as the average lifetime of the excited state (≈100 µs; Supplementary Fig. 7), the quantum yield (≈0.1^28^) of bulk EB, the efficiency of the optical setup (see Supplementary Methods for an accurate calculation and Supplementary Table 1), it was thus possible to estimate the number of luminescent centers *N* (122–1250).

From the crystal structure of EB, it follows that there are 3.8 Cu^2+^ ions nm^−2^ of a single-layer EB-NS^43^ (Supplementary Fig. 2). If every Cu^2+^ ion serves as luminescent center, these numbers would be in agreement with around 6–18 nm large (squared) single-layer EB-NS. From a resolution-limited EB-NS image, we cannot derive the lateral size and height directly. Therefore, these numbers are rather a minimum size that is in agreement with the experiment. Nevertheless, these data suggest that a large proportion, if not all, of Cu^2+^ ions act as luminescent centers. Furthermore, EB-NS showed no change of fluorescence intensity at different polarization directions (Fig. 2e) compared with a typical organic dye (Atto 488). Similar to NIR emitting lanthanide complexes, bulk EB has a long fluorescence lifetime ranging from 107 µs to 142 μs at room temperature^28,44^ and this behavior is retained in EB-NS (Supplementary Fig. 7). The long fluorescence lifetime is most likely a consequence of the parity-forbidden nature of the transition, as the local D_4h_ symmetry of the Cu^2+^ ion in EB features an inversion center^27^.

### NIR fluorescence imaging of single EB-NS

Larger EB-NS typically exhibit a layered structure as observed in scanning electron microscopy (SEM) images (Fig. 3a, b and Supplementary Fig. 8). PB milling followed by exfoliation via tip sonication enabled us to continuously control the decrease in size of EB from large macroscopic particles to micrometer large structures down to EB-NS. These large and layered EB-NS can be imaged by a NIR fluorescence microscope, but do not show a uniform fluorescence intensity profile, indicating that differences in thickness or geometry might affect fluorescence emission intensities. EB-NS smaller than the resolution limit of light microscopy can also be imaged in the NIR (Fig. 3c, d, e). To prove this point, both a Si-based and an InGaAs camera were employed in a wide-field setup. The laser intensities in this setup corresponded to the left region (<10 µW) in the confocal setup used for saturation experiments (Fig. 2d). Si-based cameras have the highest quantum yield in the visible range and not the NIR, which limits the possible speed of imaging to around 50 fps (Fig. 3c, d and Supplementary Fig. 9). In contrast, with an InGaAs camera, resolution-limited EB-NS could be imaged at video rates up to 200 Hz (Fig. 3e). These images clearly demonstrate that the NIR fluorescence properties of macroscopic EB particles are retained in nanosheets.

### Correlation between size and fluorescence intensity of EB-NS

The previous observations raise the question of how fluorescence intensity scales with size and if there is a lower limit, which is also relevant for potential bioimaging applications. Unfortunately, NIR microscopy is not able to directly assess the EB-NS size below the resolution limit. The resolution limit of light microscopy is described by the Abbe law (*λ*/2 *≈* 910 nm/2 *≈* 450 nm) and an exemplary resolution-limited single EB-NS is shown in Fig. 3e. The photon counting experiments from Fig. 2d show that there are many luminescent Cu^2+^ centers in one EB-NS, but because of the resolution limit it is not possible to assess the actual size. To provide an unambiguous answer, a correlative method that measures both size and fluorescence intensity of the same single nanosheet is required.

To understand whether and how nanomaterial fluorescence intensity is affected by size, we performed two independent correlative analyses: (1) dual SEM-NIR fluorescence imaging and (2) single-particle tracking of EB-NS in a viscous glycerol solution. For the first approach, EB-NS were deposited on a glass cover slide with a grid. The labeled meshes enabled us to image the same nanostructures both under our NIR fluorescence imaging setup and under the SEM. As shown in Fig. 4a, it was thus possible to further confirm that diffraction-limited particles <200 nm (long axis) can be imaged with our protocol. Smaller EB-NS could not be found in the SEM most likely because the contrast of a thin silicate nanosheet on glass (SiO_2_) is too low and the material is non-conducting. However, scanning transmission electron microscopy (STEM) images showed EB-NS down to few tenths of nanometer in agreement with the AFM data (Supplementary Fig. 10).

In the second correlative approach, single EB-NS were imaged and their fluorescence intensity quantified in a viscous glycerol solution (chosen to slow down diffusion). This method allowed us to simultaneously quantify fluorescence intensity and Brownian motion as a measure of size of the same EB-NS. For this purpose, the particle trajectories were tracked and the mean square displacement (MSD, 〈**r**^2^〉) determined. We used the maximum value of the total integrated brightness of the tracked blob along each trajectory (“mass”) as a measure of fluorescence intensity, to account for out-of-focus movement or rotations. A size equivalent can be derived from the trace of the random walk (Brownian motion) by employing the Stokes–Einstein equation. In Supplementary Figs. 11 and 12, trajectories and corresponding MSDs and NIR fluorescence images of exemplary EB-NS are shown. The trajectories (see Supplementary Fig. 11) show that the brighter particles move slower, indicating again that fluorescence intensity depends on size. To further investigate this finding, MSD curves were plotted and fitted: to estimate the EB-NS size, the fit was extended up to a certain lag time *τ* (25 s) and the diffusion constant (*D*)

2 $$D = \frac{\langle{{r}}^{2}\rangle}{{4\tau }}$$

was calculated. The Stokes radius *R* is related to the diffusion constant via the Stokes–Einstein equation:

3 $$R = \frac{{k_{\mathrm{B}}T}}{{6{\uppi}\eta D}},$$

where *η* is the dynamic viscosity of the solvent (Supplementary Fig. 13), *T* the temperature (Supplementary Fig. 14), and *k*_B_ the Boltzmann constant. As EB-NS are rather anisotropic with a large aspect ratio (Figs. 1 and 3, and Supplementary Fig. 8), one could assume that diffusion is dominated by the diameter and not the height similar to a spherical particle. Approximations that correct the Stokes–Einstein equation for anisotropy have been used, e.g., to analyze carbon nanotube diffusion and length^45^. Similarly, we approximated the EB-NS by a nanometer-sized spheroid, inspired by the work of Happel et al.^46^, and confirmed the results by COMSOL simulations (see Supplementary Methods and Supplementary Fig. 15 for details). We calculated a correction factor of *A* = 1.49 and corrected the results obtained from the Stokes–Einstein by this value leading to larger hydrodynamic radii. Using this method, both fluorescence intensity and hydrodynamic radii of single EB-NS are accessible (Fig. 4b and Supplementary Figs. 16 and 17).

The sizes found in Fig. 4b are in agreement with the AFM-derived sizes in Fig. 1 but represent the (larger) hydrodynamic radii and not the actual physical particle radii (Supplementary Fig. 11). The results show again that larger EB-NS are brighter than smaller ones, indicating that fluorescence intensity correlates with the number of luminescent Cu^2+^ centers and thus the volume. The spread of data points along the fluorescence intensity axis at a given Stokes radius is most likely due to different layer numbers for sheets of similar diameter (and the already discussed out-of-focus issue as well). A power-law fit shows that fluorescence intensity scales with the square root of the diameter (Supplementary Fig. 16). However, future purification approaches that lead to samples of precise layer number and sheet diameter could increase the experimental accuracy and further enhance our understanding of how EB-NS fluorescence scales with dimension. Together with the photon counting experiments in Fig. 2, these results indicate that even the smallest EB-NS are fluorescent and fluorescence emission intensity correlates with the number of Cu^2+^ ions. Therefore, dimensionality does not appear to affect per se fluorescence properties beyond the change in the number of light-absorbing and -emitting Cu^2+^ centers.

### Single-particle tracking of EB-NS in *Drosophila melanogaster* embryos

**Fig. 5:**
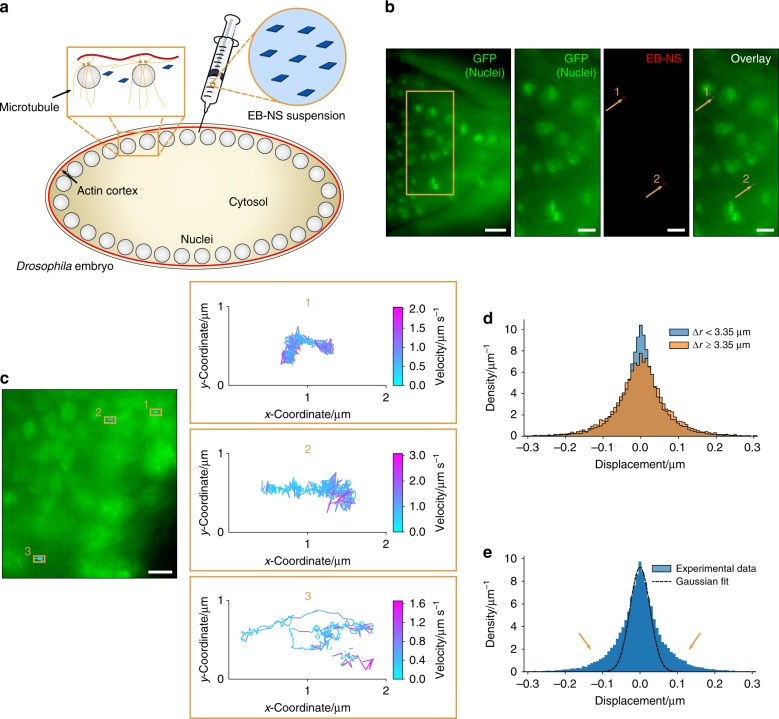
In-vivo single-particle tracking and microrheology with EB-NS. **a** Experimental scheme for microinjection of EB-NS into syncytial *Drosophila* embryos. During the syncytial blastoderm stage, the peripheral nuclei are linked to the actin cortex of the plasma membrane (red). **b** EB-NS (red) inside a *Drosophila* embryo during gastrulation stage (stage 6) expressing Histone2Av-GFP (green) that labels the nuclei. Overview and magnified region (orange rectangle) with GFP (green), NIR EB-NS (red), and overlay channels are shown. Arrows pinpoint to the two EB-NS in this field of view. Scale bar = 10 µm (overview) and 5 µm (other channels). **c** Color-coded trajectories representing the instantaneous velocity of three tracked nanosheets moving in the space between the nuclei (lag time = 0.1 s). Particles #1, #2, and #3 have diameters (measured in the image) of 0.64 µm, 0.68 µm, and 0.52 µm, and are most likely resolution-limited. Scale bar = 5 µm. **d** Van Hove histogram of displacements of two subpopulations of injected EB-NS: close to nuclei vs. distant from nuclei. The distance (Δ*r*) was computed for all points of all trajectories as the minimum distance to the border of any nucleus. **e** Van Hove plot of all tracked EB-NS and Gaussian fit for small displacements (range [−0.03 μm, +0.03 μm]). The tails (see arrows) of the distribution exceed the Gaussian curve, which indicates the presence of active processes in *Drosophila* that lead to larger displacements than expected from thermal motion. *n* = 38 tracked EB-NS (21 embryos).

To demonstrate the potential of EB-NS for bioimaging applications, we performed single-particle tracking and microrheology measurements of EB-NS in embryos of the fruit fly *D. melanogaster*. This fly species is a widely employed model organism for studies ranging from fundamental genetics to developmental cell biology. During embryonal development the nuclei arrange in complex patterns mediated by microtubules and the actin cortex, but the underlying mechanisms and mechanics are poorly understood^47^. Following fertilization, the embryo develops as a syncytium^47^ in which the nuclei arrange in a regular 2D array linked to the actin cortex of the plasma membrane (Fig. 5a).

The dynamics of the nuclei and their associated centrosomes is determined by the cortical link and internuclear interactions mediated by microtubules^48^. It has been hypothesized that the viscoelastic properties and the molecular motors play a key role in this process^47^. To elucidate the micromechanical properties of the cellular matrix, we introduced EB-NS into *Drosophila* embryos and tracked single EB-NS via NIR fluorescence microscopy. Imaging in *Drosophila* is challenging due to autofluorescence in the visible region and its high sensitivity to phototoxicity; therefore, NIR imaging would be very beneficial.

For assessing the relative location of the EB-NS in *Drosophila*, mutants expressing green fluorescent protein (GFP)-labeled histones in their nuclei were used (Fig. 5b). Using this approach, we followed the traces of single EB-NS in vivo (Fig. 5b), allowing long-term imaging without bleaching or developmental abnormalities despite the high temporal resolution with frame rates of 10 Hz. In addition, the low autofluorescence in this spectral range enabled a higher contrast of the NIR EB-NS image compared to the visible GFP image (Fig. 5b). These results highlight the great advantages of a non-bleaching NIR nanoscale fluorophore compared with typical organic dyes. To further prove the potential of EB-NS as NIR fluorophore for bioimaging, we were inspired by recent studies in which NIR fluorescent carbon nanotubes were used to explore the extracellular space between neurons^49^. The instantaneous velocity of the injected EB-NS moving between nuclei was therefore analyzed (Fig. 5c and Supplementary Fig. 18). The goal was to use EB-NS as a probe for the extracellular space as a function of the distance to nuclei. To best display our results, established analysis tools such as the Van Hove histogram of displacements^50^ were chosen (Fig. 5d). The analysis revealed two subpopulations. EB-NS closer to nuclei showed smaller displacements, indicating a denser environment around the nuclei. EB-NS are likely to probe the mesh-size of the embryo’s dense cytosol on the nanoscale and report mechanical properties on a different size scale than typical micrometer-sized beads used for microrheology^47^. Inside the embryo, one can expect active processes such as flow or motor-driven contractions (“active matter behavior”). Van Hove histograms provide access to test if there are such processes (Fig. 5e). The tails of the distribution go beyond the Gaussian function fit (thermal motion) and indicate the presence of active processes^50^. These could be, e.g., microtubules and kinesin motors (in the spindle close to the nuclei) that move the nuclei apart from each other and thereby lead to larger displacements of nearby EB-NS. Our results therefore show that EB-NS are powerful probes for studies of biological systems.

### Biocompatibility and stand-off detection in living plants

In the context of biological applications, toxicity is a major concern and, e.g., a potential drawback of quantum dots that contain toxic elements and 2D materials in general^51,52^. To evaluate cytotoxicity, viability assays with EB-NS exposure to different cell lines (A549, 3T3, and MDCK-II) were performed. We observed no significant effects on the viability of these cell lines, highlighting the biocompatibility of EB-NS (Supplementary Fig. 19). In contrast, Cu^2+^ from soluble CuSO_4_ decreased cell viability, which shows that EB-NS does not release relevant Cu^2+^ ion concentrations as expected from a stable silicate. Overall, this study demonstrates that EB-NS are powerful and biocompatible nanoprobes in living organisms for single-particle tracking and microrheology measurements in the transparent NIR window.

Another potential application of NIR fluorescent EB-NS is non-contact stand-off detection of nanoprobes in living organisms with minimal perturbation. The low autofluorescence in the NIR region provides a high contrast, but stand-off detection requires especially bright fluorophores, because only a small portion of emitted light reaches the detector. Recently, NIR fluorescent SWCNT sensors enabled plants to report to electronic devices chemical signaling processes, pollutants in the environment^53^, and explosives^54^. Optical nanosensors are poised to allow the engineering of smart plants that communicate with and actuate agricultural and phenotyping devices for improving crop productivity and resource use^24^.

**Fig. 6:**
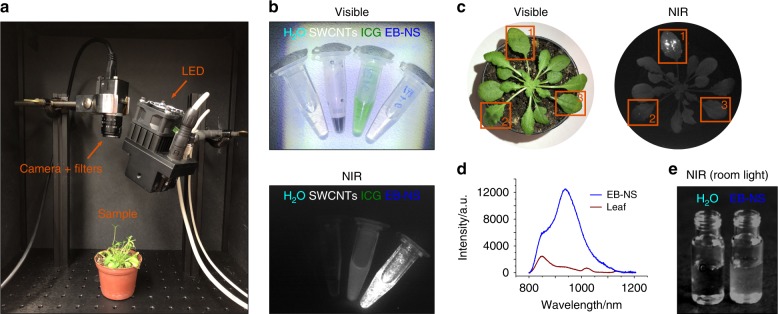
Stand-off detection of EB-NS fluorescence in plants through low-cost and widely available imaging devices. **a** Photograph of a plant of *Arabidopsis thaliana* placed in a low-cost stand-off imaging system, which consisted of a LED, NIR filters and a Si-CMOS camera. **b** Visible and NIR fluorescence images of EB-NS (≈0.1 mg mL^−^^1^) compared with other NIR nanomaterials and fluorophores at similar concentration (single-walled carbon nanotubes (SWCNTs), indocyanine green (ICG)). Water is used as negative control. **c** Visible and NIR images of an *Arabidopsis* plant, which was infused with EB-NS (frame 1), SWCNTs (frame 2), and buffer only (frame 3). **d** The NIR fluorescence spectrum of the leaf confirms the presence of EB-NS and its strong emission compared to the leaf background. Both results demonstrate the high brightness of EB-NS compared to state-of-the-art NIR nanomaterials (SWCNTs) and that this platform can be applied for stand-off detection using a low-cost optical setup. **e** The EB-NS emission can even be detected without LED excitation under room light conditions.

Herein, we developed a low-cost optical setup to image the NIR emission of EB-NS from a distance >10 cm and compared it with several other NIR fluorophores. The setup consisted of a white light LED and a camera (1280 × 1024 pixels) equipped with NIR (900–920 nm) long-pass filters (Fig. 6a) corresponding to 76 μm pixel^−1^. The high brightness of EB-NS allowed us to use a simple CMOS camera (Complementary Metal Oxide Semiconductor, Si-based) instead of expensive electrically or nitrogen-cooled InGaAs cameras (>40k €), which would have a much higher quantum yield in this spectral range. Quantum yield of Si-based cameras decrease with increasing wavelength and therefore this low-cost setup favors fluorophores with lower emission wavelength. Therefore, ICG should appear slightly brighter and SWCNTs slightly dimmer than they really are and EB-NS would be in the middle. However, the SWCNT sample also contained (6,4)-SWCNTs that emit at 870 nm. These different experimental conditions should be taken into account when assessing the performance of this low-cost setup. In the visible image (Fig. 6b), one can easily distinguish SWCNTs (black) from ICG (green), whereas the solution with EB-NS is transparent similar to the water control.

In contrast, in the corresponding NIR fluorescence image (Fig. 6b), EB-NS are significantly (2×) brighter than ICG and 10× brighter than SWCNTs (Fig. 6b and Supplementary Fig. 20). Interestingly, stand-off detection of the NIR emission of EB-NS is also possible without LED excitation: room or sunlight alone is sufficient to generate detectable NIR fluorescence from EB-NS (Fig. 6e).

Buffered EB-NS were infused into the leaves of *Arabidopsis thaliana*, a well-established plant model system (Fig. 6c, d)^53^, using a standard method for nanomaterial delivery into plant leaves in-vivo^53^. The same approach was used for the infiltration of SWCNTs in separate leaves in the same plant, which served as a visual comparison with EB-NS. As it can be observed from the NIR image in Fig. 6c, plant leaves are characterized by a background autofluorescence in this wavelength region that hinders the visualization of SWCNTs fluorescence (frame 2) through a Si-based CMOS camera, whereas EB-NS can be detected with a strong fluorescence signal (frame 1). Similarly, fluorescence spectra of these leaves (Fig. 6d) show the strong EB-NS emission compared to the (autofluorescence) background. These stand-off detection experiments highlight that EB-NS can be delivered into living organisms with high background fluorescence such as plants and can be detected using low-cost and widely available stand-off imaging devices.

## Discussion

Our results show that EB-NS have useful properties for NIR bioimaging applications including ultra-high photostability, high brightness for NIR fluorophores, and biocompatibility. The remarkable properties of EB-NS are retained even when the dimensions of the nanosheets are reduced. Our data indicates that most if not all copper ions serve as luminescent centers that emit NIR light. It only affects the absorption cross-section by changing the number of light-absorbing Cu^2+^ ions. In contrast, the quantum yield of NIR fluorescent SWCNTs increases with length. As a consequence, shorter SWCNTs (<100 nm) are much less bright than longer ones^40^. Therefore, SWCNTs cannot be shortened without disadvantages. Although it has been reported that for bioimaging applications the NIR-II (1000–1700 nm) windows further improves tissue penetration^5^, a major advantage of the EB-NS fluorescence in the NIR-I region (800–1000 nm) is the wide availability of cameras^55,56^. Instead of expensive (>40k €), liquid nitrogen-cooled, low-resolution InGaAs-based cameras, low-cost, (1k €) high-resolution, Si-based cameras can be used for detection. In the past, (6,4)-SWCNTs with smaller diameter that emit in the NIR-I region at 870 nm have been isolated exactly for this purpose^57^. Even though the quantum yields of Si-based cameras rapidly decrease in the NIR (around 5% >900 nm), they are still able to image EB-NS using a normal microscope or a low-cost stand-off detection device. Furthermore, there exist (bulk) pigment homologs Han Blue (BaCuSi_4_O_10_) and Han Purple (BaCuSi_2_O_6_), which have a red-shifted emission spectrum up to 1000 nm, and they can be exfoliated into nanosheets using the same protocol as for EB (Supplementary Fig. 21). The capability of imaging EB-NS even with only room light excitation using Si-based cameras is particularly relevant for commercial applications beyond the well-equipped research laboratories or biomedical research, e.g., the engineering of smart plants for high-throughput phenotyping and precision agriculture. Another major advantage of EB-NS is their low toxicity, proven by the performed viability assays on cells (Supplementary Fig. 19). With our study, we add EB-NS to the library of 2D materials in general and especially exfoliated silicates and clays^58,59^. In the future, EB-NS and their physicochemical properties could be further investigated to find non-expected properties as in other 2D materials such as transition metal dichalcogenide nanosheets^60^. In addition, the surface chemistry could be tailored with biomolecules similar to silica nanoparticles and silicate-coated core-shell nanoparticles^61,62^.

In summary, we developed a method to exfoliate the calcium copper silicate EB into nanosheets (EB-NS) and demonstrated that their NIR optical properties are retained at the nanoscale with copper ions as luminescent centers. We add a promising 2D material to the class of NIR fluorophores with ultra-high photostability, high brightness and biocompatibility. EB-NS have a large potential for demanding biological applications such as in-vivo imaging in complex biological matrices, including animal and plant model systems.

## Methods

### Exfoliation of Egyptian Blue

EB powder was purchased from Kremer Pigmente GmbH & Co. KG., and EB-NS were prepared as follows. If not stated otherwise, a first milling step was carried out. To produce sufficient amounts of nanoparticles, we used a PB mill (PB, Pulverisette 7 Premium Line, Fritsch, Germany) equipped with 20 mL agate beakers and 5 mm agate balls. The milling was performed in deionized water at 900 r.p.m. for up to 60 min. The resulting grainsize distribution was analyzed by laser diffraction carried out with a LS13320, Beckman&Coulter laser particle sizer (Germany). Three runs after a short tip sonication step were done. PIDS was used and an optical model R.I. 1.6/1 was applied. The so-generated and characterized EB slurry was afterwards allowed to settle down overnight and then decanted: only the supernatant portion of the slurry was dried and the so-obtained powder (visibly brighter, a consequence of the dichroism of EB^25^) was finally processed with a long-tip sonication step. In more detail, 10 mg of EB PB-milled powder was transferred to a glass vial together with 5 mL of isopropanol (Fischer Chemical, 99.98%) or milli-Q water. Tip sonication was performed in a glass vial with a Fisherbrand™ Model 120 Sonic Dismembrator (Fischer Scientific) in an ice bath for 1–6 h at 60–72 W (if not stated otherwise: typical tip sonication time and power percentage were 6 h and 60%, respectively). EB dispersions were stored at room temperature and vortexed prior to use (Vortex Mixer VV3, VWR International) for 10 s at maximum power (2500 min^−1^, 10 W).

### Atomic force microscopy

For this dataset, no PB milling step prior to tip sonication was performed. The pristine powder was ground with mortar and pestle till a visible color change could be observed, then it was tip sonicated (in isopropanol) as described in the previous section. One hundred microliters of EB-NS stock dispersion (6 h tip sonicated) were diluted 10× with isopropanol and vortexed for 10 s. Ten microliters of EB-NS were spin-coated (G3 Spin Coater, Specialty Coating Systems, Inc.) on a mica surface. The substrate was kept spinning at 500 r.p.m. (7 RCF) for 2 min (5 s of ramp time, 30 s of dwell time). An Asylum Research MFP-3D Infinity AFM (Oxford Instruments) was employed in AC mode (software version 15.01.103). Rectangular cantilevers from Opus (160AC-NA, MikroMasch Europe) were used (aluminum coating, tetrahedral tip, 300 kHz resonance frequency, force constant of 26 N m^−1^). Image analysis was performed with Gwyddion (version 2.51).

### NIR fluorescence spectroscopy

The setup consists of a monochromator (MSH-150, LOT-Quantum Design GmbH) equipped with a xenon arc lamp and a diffraction grating, an Olympus IX73 microscope with a 10× objective (UplanFLN 10×/0.30, Olympus), and a Shamrock 193i spectrograph (Andor Technology, Ltd) coupled to an array NIR detector (Andor iDUs InGaAs 491). Spectra were recorded from EB-NS dispersions at an excitation wavelength of 615 nm with an exposure time up to 5 s and a slit width up to 500 µm. The Andor SOLIS software (version 4.29.30012.0) was employed for the acquisition of spectra, which were and further analyzed with Origin Pro 8.1. For 2D spectra, EB-NS were placed on glass substrates. The excitation wavelength was scanned in 4 nm steps with the monochromator, and at each wavelength a spectrum was recorded with an integration time of 1 s at a slit width of 10 µm. The 2D spectra were corrected for the quantum efficiency of the detector and the spectral irradiance of the xenon lamp of the monochromator using a self-written Python script.

### Fluorescence saturation measurements

The main components of the employed setup were a laser source (Supercontinuum laser SC400-4-20, Fianium), a photodetector (single photon avalanche diode PDM series, MPD), and a 60× objective lens (Apo N, 60×/1.49 numerical aperture (NA) oil immersion, Olympus). For this dataset, no PB milling step prior to tip sonication was performed. The pristine powder was ground with mortar and pestle till a visible color change could be observed, then it was tip sonicated (in isopropanol) as described above. Ten microliters of the supernatant of a 6 h tip-sonicated EB-NS sample (1:100 diluted in isopropanol) were spin-coated on a glass cover slide. Spin-coating parameters were the same ones chosen for AFM measurements. Despite the polydispersity of EB-NS, scanning them using a confocal microscope through the diffraction-limited focal spot of 1.49 NA objective allowed us to select only the smallest particles with sizes estimated to be not exceeding the dimensions of the focal spot. The spot size of larger EB-NS exceeded the dimensions of a diffraction-limited focal spot, thus allowing us to distinguish them from smaller particles. After selecting a particle, we measured its fluorescence intensity at different excitation powers. Excitation of nanoparticles that have relatively long excited state lifetimes of the order of 100 µs (Supplementary Fig. 7) with a laser that has high repetition rate (20 MHz) allows one to extrapolate saturation of the nanoparticle’s luminescence. The solid circles in Supplementary Fig. 6a show the dependence between the brightness of the particle and the excitation power that was measured directly before the objective lens. The excitation wavelength was 640 nm. The fluorescence intensity values were normalized to the detection efficiency of the microscope, taking into account the average collection efficiency of light by the 1.49 NA objective lens (70%, given the random orientation of the emission transition dipoles), transmissivity of all the optical elements involved (~50%) and detection efficiency of the single photon avalanche diode (PDM series, MPD) in the current spectral range (~5%). It is noteworthy that as a result of the above normalization, luminescence intensity in Fig. 2d corresponds to the total number of photons emitted by the particle.

The experimental data were fitted with a typical fluorescence saturation function^63^:

4 $$I = I_{{\mathrm{sat}}}\frac{{P_{{\mathrm{exc}}}}}{{P_{{\mathrm{exc}}} + P_{{\mathrm{sat}}}}},$$

where *P*_exc_ is the excitation power, *P*_sat_ is the saturation power, *I*_sat_ is the nanoparticle luminescence intensity that can be detected at the saturation excitation power. This function yields the values of *P*_sat_ and *I*_sat_ for every saturation curve measured. Supplementary Table 1 shows the obtained values.

The obtained saturation luminescence intensity values allow one to estimate the number of luminescent centers *N* per particle. Taking into account the average excited state lifetime *τ* (~100 µs, Supplementary Fig. 7) and quantum yield *Φ* (~0.1^28^), we recalculated the number of emitted photons *I*_sat_ per second into the number of centers:

5 $$N = \frac{{I_{{\mathrm{sat}}}}}{{{\it{\Phi}} \tau ^{ - 1}}}.$$

### Fluorescence polarization measurements

The setup mainly consisted of a light source (LED, Lumencor), which emitted unpolarized excitation light, a photodetector (EMCCD camera, iXon Ultra DU-897U-CS0, Andor) and a 60× objective lens (Apo N, 60×/1.49 NA oil immersion, Olympus). Measurements were performed by recording fluorescence images of individual EB-NS at different positions on a linear polarizer, which was placed in front of the camera. The sample preparation steps coincided with the ones performed for fluorescence saturation measurements. Atto 488, purchased from Atto-Tec, was used as a reference.

### NIR fluorescence imaging setup

An Olympus BX53 microscope equipped with 20× (MPlanFL N 20×/0.45, Olympus) and 100× (UPlanSApo 100×/1.35 Sil, Olympus) objectives was used. A Xeva-1.7-320 NIR camera (Xenics®) and a Zyla 5.5 sCMOS camera (Oxford Instruments) were used to observe the EB-NS fluorescence excited by a 561 nm laser (Cobolt Jive^TM^ 561 nm). Typically, a droplet of an EB-NS dispersion was put on glass slides, dried and imaged at 10–50 mW excitation power. To assess bleaching, a solution of Rhodamin B (Sigma-Aldrich) in isopropanol was prepared and a drop of this solution was put on a glass slide for imaging. Dried EB-NS were put on a separate glass slide. At a continuous excitation of 100 mW at 561 nm, images with an integration time of 100 ms were recorded 120 times every 0.5 min in the case of Rhodamin B and every 1 min with an integration time of 1 s in the case of EB-NS. For Rhodamin B the Zyla camera was used and for EB the Xenics NIR camera was used. The specified fluorescence intensity corresponds to the mean gray value of the images.

For video-rate imaging and size-fluorescence correlation experiments, a modified setup equipped with a Cheetah 640TE3 camera (Xenics NV, Belgium) was used for NIR detection. Here, the light was passed through a dichroic mirror (HC BS R785, AHF, Germany) and a long-pass filter (FELH0900, Thorlabs, Inc., USA) before reaching the camera’s sensor. Images were captured with the 100× objective.

### Video-rate imaging of EB-NS with an InGaAs camera

EB-NS (previously milled and tip sonicated in isopropanol) underwent a single centrifugation step (240 × g for 2 h) to remove unexfoliated material. Ten microliters of EB-NS sample were spin-coated onto a #1 glass coverslip with the following settings: 1000 r.p.m. (14 RCF) for 1 min (40 s of ramp time, 20 s of dwell time). The sample was then placed under the NIR imaging setup and pictures were taken with a 100× objective, laser power of 500 mW and exposure times in the range from 1 s to 5 ms (corresponding to frame rates ranging from 1 to 200 fps). Data analysis was performed with ImageJ (v. 1.52a) and Origin Pro 8.1 software.

### Scanning electron microscopy and SEM-NIR correlation

For a first qualitative observation of exfoliated EB-NS (Fig. 3a, b), no PB milling step prior to tip sonication was performed. The pristine powder was ground with mortar and pestle till a visible color change could be observed, then it was tip sonicated (in isopropanol) as described above. Ten microliters of EB-NS suspension were drop-casted on a Si wafer. Both gold sputtering and evaporation (≈2 nm of gold layer) were tested on different samples; nevertheless, the best imaging conditions were met when no gold deposition step was performed at all. Interestingly, EB was conductive enough to be seen at SEM without gold deposition. This sample was observed under a LEO SUPRA 35 microscope (Zeiss) with an Inlens detector at 20 kV (secondary electrons).

For the SEM-NIR correlation, EB-NS (previously milled and tip sonicated in isopropanol) were centrifuged (240 × *g* for 2 h). This way unexfoliated (bulk) material could be removed. Meanwhile, a #1.5 gridded glass coverslip (Gridded Glass Coverslips Grid-50, Ibidi, Germany) was stirred for 20 min at 70 °C in a 5 : 1 : 1 solution of milli-Q water : hydrogen peroxide : ammonia. Afterwards, the substrate was dried with a nitrogen gun and, to increase the adhesion of EB-NS on its surface, plasma treatment (Zepto, Diener Electronic GmbH +Co. KG, Germany) was performed: this step included 1 min of O_2_ supply and 1 min of plasma process. After cleaning the substrate as described, 10 μL of EB-NS sample were spin-coated onto it with the following settings: 1000 r.p.m. (14 RCF) for 1 min (40 s of ramp time, 20 s of dwell time). The prepared sample was then observed at the NIR imaging setup (see corresponding section for more info) with a 100× objective, 1 s of exposure time, and 500 mW of laser power. Finally, the substrate was moved to the Quattro S SEM setup (Thermo Fisher Scientific, USA) employed for the size-fluorescence correlation analysis. Typical imaging settings here were: low vacuum mode, LVD detector (Thermo Fisher Scientific, USA), high voltage = 15 kV (secondary electrons), chamber pressure = 1.25 mbar (water), high voltage = 15 kV, and spot size = 3.5. Data analysis was performed with ImageJ (v. 1.52a) and Origin Pro 8.1 software.

### Correlative size-intensity measurements in glycerol

EB-NS (previously milled and tip sonicated in water) were size-selected by means of two steps of liquid cascade centrifugation:^33,64^ in this way, we could get rid of unexfoliated EB and increase the monodispersity of the pellet obtained after the last step (first centrifugation = 240 × *g* for 2 h, second centrifugation = 2660 × *g* for 2 h). Glycerol (0.5 mL) (Alfa Aesar, 99+%) was then added to the dried final pellet (≈1 mg) and the sample was finally tip sonicated in an ice bath for 2 min at 60% to achieve a homogeneous redispersion. For imaging, 10–20 µL of the glycerol sample were introduced into a flow chamber (µ-Slide VI 0.5 Glass Bottom, Ibidi, Germany) and placed under our NIR imaging setup. A 100× objective was employed to observe EB-NS at 8 fps (≈50 ms exposure time, 1000 frames) with a laser power of 250 mW, which, as shown in Supplementary Fig. 14, was not overheating the sample significantly during acquisition. Particle tracking and MSD calculations were performed using a self-written Python script based on the Trackpy package^65,66^ (v0.4.2 on Python v3.7.3^67^), whereas the final steps of data analysis were performed on Origin Pro 8.1 software. For identification and linking of particles into trajectories, the following parameters in Trackpy were implemented: memory = 300, minimum number of points (i.e., trajectory length) = 300, search_range (i.e., maximum displacement between consecutive frames) = 9, diameter (of the tracked blob) = 11. The tracking of the Brownian motion of an EB-NS yields the trajectory of the particle as a set of time-dependent *x* and *y* positions for *N* time steps of length *τ*. From the *x* and *y* positions, the square displacement for the *n*-th time step ${\mathbf{r}}_n^2$ is calculated as

6 $${{r}}_n^2 = \left( {{{x}}\left( {n \cdot \tau } \right) - {{x}}\left( {\left( {n - 1} \right) \cdot \tau } \right)} \right)^2\, +\, \left( {{{y}}\left( {n \cdot \tau } \right) - {{y}}\left( {\left( {n - 1} \right) \cdot \tau } \right)} \right)^2,$$

where *n* ranges from 1 to *N*. The MSD 〈**r**^2^〉 is simply the mean over all single time step values:

7 $$\langle {\mathbf{r}}^{2}\rangle = \frac{1}{N}\mathop {\sum }\limits_{n = 1}^N {\mathbf{r}}_n^2.$$

Assuming the diffusion is restricted to two dimensions, the diffusion coefficient *D* and the MSD are linked via the relation

8 $$D = \frac{{{{r}}^2}}{{4\tau }}.$$

MSD curves were fitted up to a maximum lag time of 25 s, to measure the diffusion coefficient of each particle. Finally, from the Stokes–Einstein equation, the Stokes radius *R* is calculated as

9 $$R = \frac{{k_{\mathrm{B}}T}}{{6{\uppi}\eta D}},$$

where *η* is the dynamic viscosity of the solvent (Supplementary Fig. 13), *T* the temperature (Supplementary Fig. 14), and *k*_B_ the Boltzmann constant. The Stokes–Einstein equation is only strictly valid for spherical particles. Due to the high anisotropy of EB-NS, we assumed that Brownian motion is dominated by the diameter of the nanosheets and not the much smaller height.

### In-vivo microrheology of *D. melanogaster* embryos

EB-NS (previously milled and tip sonicated in water) were filtered with a 0.20 μm syringe filter. To further enhance the concentration of the smallest nanosheets in the sample, the filtered sample vial was placed in a concentrator (Eppendorf® centrifugal vacuum concentrator, Eppendorf, Germany) for 45 min at 45 °C and finally bath-sonicated for 10 min to reduce agglomeration. *D. melanogaster* embryos expressing Histone2Av-GFP^68^ with an age of 0–1 h were collected and dechorionated with hypochlorite for 120 s, washed with water thoroughly, aligned on a piece of agar, transferred to a coverslip coated with glue and covered with halocarbon oil (Voltalef 10 S, Lehmann & Voss) after a slight desiccation. An aliquot of suspension in water of the EB-NS sample was injected using Microinjector FemtoJet® (Eppendorf) on an inverted microscope: the injection volume is calculated according to literature^69^. After around 30 min of incubation, the sample could be moved to the NIR setup mentioned above for colocalization experiments. EB-NS were excited with the 561 nm laser (up to 500 mW), whereas a fluorescence lamp (X-Cite® 120Q, Excelitas Technologies) was used for GFP excitation. Both channels were observed through a 100× objective and recorded with a Zyla 5.5 sCMOS camera. An exposure time of 0.1 s was chosen and images on both channels were taken for 60 s (10 fps). A 2 × 2 pixel binning was performed to lower the size of acquired data and thus facilitate the following particle tracking analysis. As for the EB-NS tracking in glycerol, custom-made scripts based on Trackpy were employed to analyze the acquired frames. Only the EB data were evaluated, since the nuclei did not display any significant motion during the acquisition. Typical parameters employed for particle tracking were: memory = 300 frames (features vanishing for more than 300 frames were considered separate particles), minimum number of data points = 200, maximum displacement between frames = 9 pixels, and diameter (of the tracked blob) = 11 pixels. The perimeter of the nuclei was manually measured on ImageJ and its coordinates implemented in a self-written Python code which could assess the minimum distance of each point along a trajectory to the closest nucleus perimeter. With a separate code, instantaneous velocity plots (lag time = 0.1 s) could be evaluated and plotted. Van Hove histograms were plotted in Python, too, inspired by past works in literature^50^. The overlying Gaussian fit was performed for small displacements (range [−0.03 μm, +0.03 μm]).

### Stand-off detection

NIR images were acquired with a CMOS-based DCC3240M camera (Thorlabs), equipped with a 900 nm (FEL0900, Thorlabs) and a 920 nm (LP920, Midwest Optical Systems) long-pass filter, mounted in series to exclude the visible background. A white light source (UHP, Prizmatix) connected to a 700 nm (FESH0700, Thorlabs) and a 750 nm (FESH0750, Thorlabs) short-pass filter was used for excitation. Exposure times between 50 and 100 ms were used for all acquisitions.

Single-stranded DNA (ssDNA)-modified SWCNTs were obtained by placing 125 µL (2 mg mL^−1^ in PBS) (AT)_15_ ssDNA (Sigma-Aldrich) and 125 µL (6,5) chirality enriched SWCNTs (Sigma-Aldrich, Product No. 773735) (2 mg mL^−1^ in PBS) for tip sonication (15 min, 30% amplitude). The obtained suspension was centrifuged twice for 30 min at ambient temperature (16100 × *g*). The amounts of ICG (≈90%, MP Biomedicals GmbH) and of a 6 h tip-sonicated sample of EB-NS in water were ≈0.1 mg mL^−^^1^.

Seeds of *A. thaliana* (Col.0 ecotype) were sown on sterilized soil (8 h, 80 °C) and stratified for 2 days in the dark at 4 °C. The plants were grown under long day length (16 h light/8 h dark) in climate-controlled growth chambers at 22 °C, 60% humidity, and light intensity of 120–150 μmol m^−^^2^ s^−^^1^. Delivery of nanomaterials and buffer into leaves of *Arabidopsis* was performed as described by Giraldo et al.^53^. In brief, 50 µl of the desired NIR fluorophore suspension were infused through the lower (abaxial) side of the leaf lamina with a needle-less syringe. Spectroscopic measurements for the presence of EB-NS in the leaf was performed using the NIR fluorescence spectroscopy setup (5 s exposure time) as described above. Regarding the imaging of EB-NS under room light excitation, 1 mL of supernatant taken from a 6 h tip-sonicated sample in water was observed. A background picture was taken and used for background subtraction.

### Reporting summary

Further information on research design is available in the Nature Research Reporting Summary linked to this article.

## Supplementary information

Supplementary Information

Peer Review File

Reporting Summary

## Data Availability

The data that support the findings of this study are available from the corresponding author upon reasonable request.

## Code availability

The codes that support the findings of this study are available from the corresponding author upon reasonable request.
