# Supplementary material for: Exfoliated near infrared fluorescent silicate nanosheets for (bio)photonics
Source: Nat Commun. 2020 Mar 20;11:1495. doi: 10.1038/s41467-020-15299-5 (PMC7083911; doi:10.1038/s41467-020-15299-5)
Supplement: Supplementary file 1 — Supplementary Information [file 41467_2020_15299_MOESM1_ESM.pdf]

Supplementary Information

# Exfoliated near infrared fluorescent silicate nanosheets for (bio)photonics

*Selvaggio et al.*

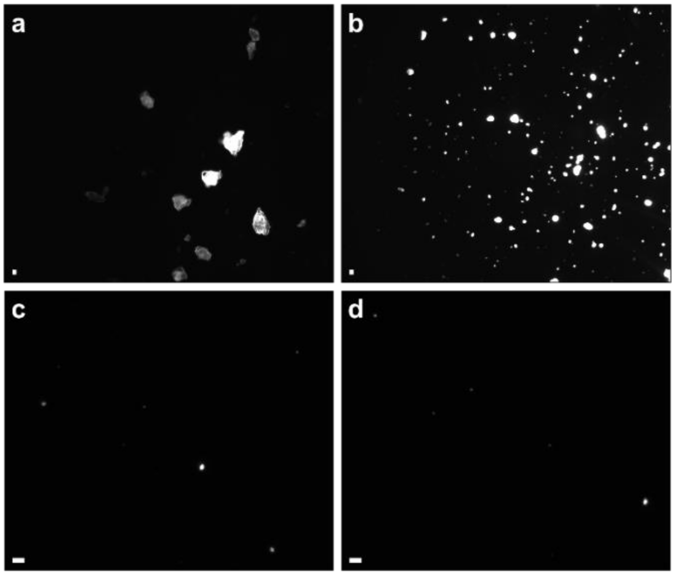


**Supplementary Figure 1** NIR fluorescence images of Egyptian Blue (EB) before and after tip sonication. **a** EB bulk powder dispersed in isopropanol. **b** Egyptian Blue nanosheets (EB-NS) after 6 h of tip sonication. **c,d** EB-NS after 6 h of tip sonication and size-cut-off filtration (*d* = 0.45 µm). All samples were drop-casted (10 µL) on glass cover slides before imaging. Every purification step reduces the overall concentration of particles but increases monodispersity. Note that the contrast was not adjusted. Therefore, the smaller EB-NS in b-d are difficult to see (compared to the larger ones) and it looks like there are not many particles left, but when zooming in they are visible. Scale bar = 10 µm.

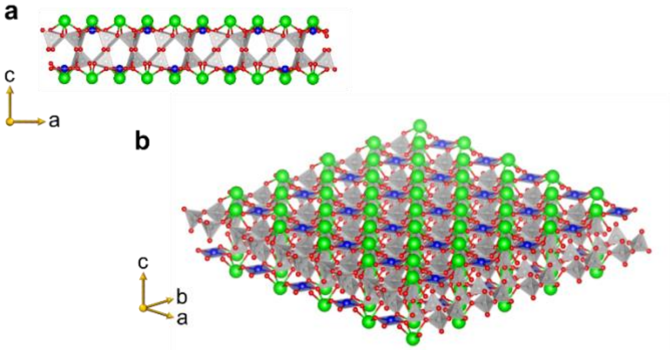


**Supplementary Figure 2** Schematic of a monolayer of EB. **a** Frontal view. **b** 3D axonometric projection. For both illustrations, EB neutron powder diffraction data obtained from literature was used[1]. Si, O, Ca and Cu atoms are depicted as gray, red, green and blue spheres, respectively. Vesta (v. 3.4.4) was employed to draw the structures[2].

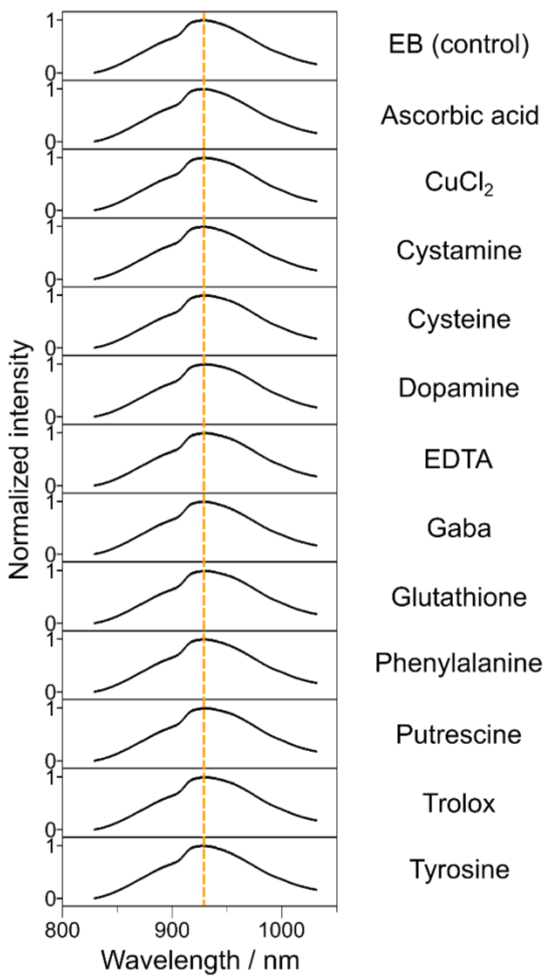


**Supplementary Figure 3** Fluorescence response of EB-NS to different analytes of interest. NIR spectra of EB-NS (6 h tip sonication, 10 µg $mL^{-1}$) in water 10 min after addition of analytes (100 µM) that are known to affect spectra of other fluorophores. The EB-NS fluorescence did not display any significant shifts. This result further shows the stable NIR fluorescence of this nanomaterial.

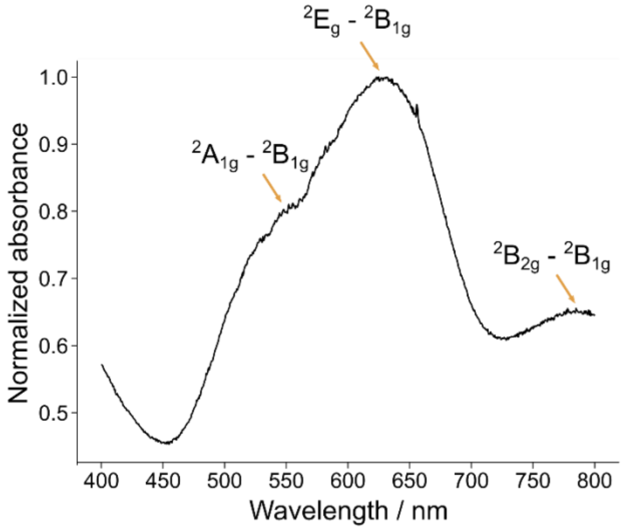


**Supplementary Figure 4** Absorption (reflection) spectrum of EB powder. Three broad bands corresponding to different electronic transitions are observed (yellow arrows). The symmetry species of the orbitals involved in the transition are indicated next to the arrows. The attribution of the bands to the symmetry species was made according to the model proposed by Accorsi *et al.*[3].

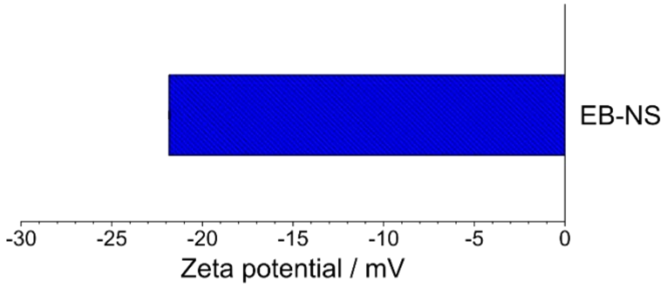


**Supplementary Figure 5** Zeta potential of EB-NS. Zeta potential of EB-NS in water (2 mg $mL^{-1}$). The error bar corresponds to the standard deviation of triplicates.

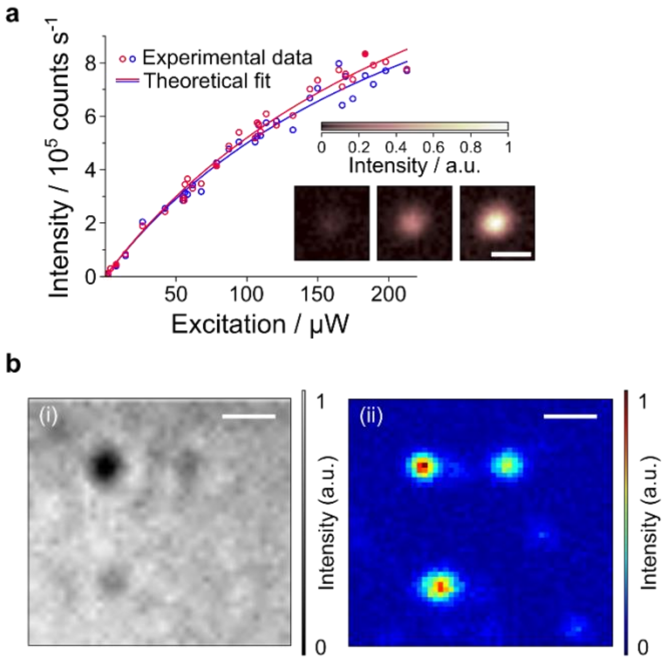


**Supplementary Figure 6** NIR fluorescence saturation measurements, white light scattering and confocal scanning fluorescence images of EB-NS. **a** Fluorescence saturation measurement of a single EB-NS. Red circles show the data measured upon initial increase of excitation power, blue circles correspond to the subsequent decrease of the excitation power; red and blue lines represent the respective fits. The inset shows three images that were acquired at different excitation powers (corresponding data points are depicted with solid circles). Scale bar = 1 µm. The obtained saturation values ($P_{sat}$) are 276 and 252 µW. The slightly lower saturation intensity value that was obtained for the process of diminishing excitation power could be caused by heating of the sample by the excitation light, which affects the fluorescence lifetime in bulk EB[3]. The obtained saturation values correspond to a fluorescence emission of ≈ 1.95 x $10^6$ photons per second. With this number we estimated the number of luminescent centers in the order of 1950 by taking into account the average lifetime of the excited state (≈ 100 µs, Supplementary Figure 7), the quantum yield (≈ 0.1[3]) of bulk EB, the quantum efficiency of the photodetector in this spectral region, the parameters of optical elements of the microscope and the acquisition time of the signal. From the crystal structure of EB it follows that there are 3.8 $Cu^{2+}$ ions $nm^{-2}$ of a single layer EB-NS[4] (Supplementary Figure 2). If every $Cu^{2+}$ ion serves as luminescent center, these numbers would be in agreement with a 23 nm large (squared) single layer EB-NS. **b** White light scattering (i) and confocal scanning fluorescence (ii) images of EB-NS taken from the same area within the same sample. Scattering intensity appears not to be proportional to fluorescence intensity. The dimmest particles in (ii) are not visible in (i) due to their reduced size. Scale bar = 1 µm.

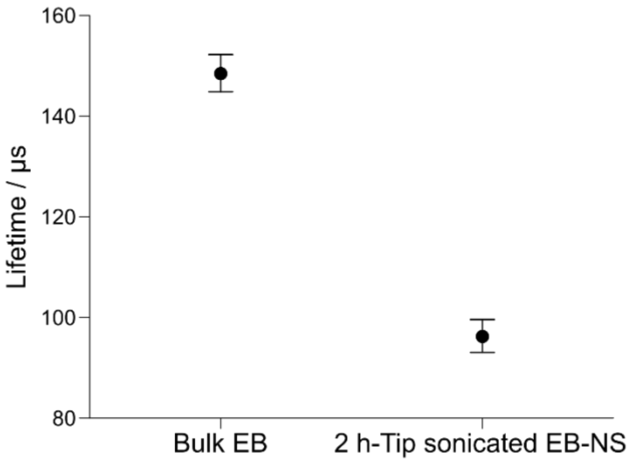


**Supplementary Figure 7** Fluorescence lifetimes of bulk EB and exfoliated EB-NS. Error bars correspond to standard deviations (*n* = 100 repetitions performed for each sample).

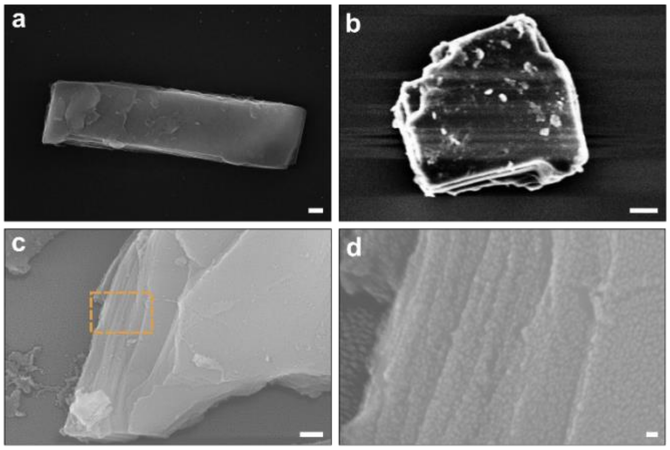


**Supplementary Figure 8** Scanning electron microscopy (SEM) images of larger exfoliated EB (nano)sheets. Different samples and preparation techniques are shown. **a** No gold deposition. Scale bar = 200 nm. **b** ≈ 2 nm evaporated gold. Scale bar = 1 µm. **c** ≈ 2 nm of sputtered gold on the surface of the sample. Scale bar = 200 nm. **d** A magnified region from the inset shown in c indicates the layered structure. Scale bar = 20 nm.

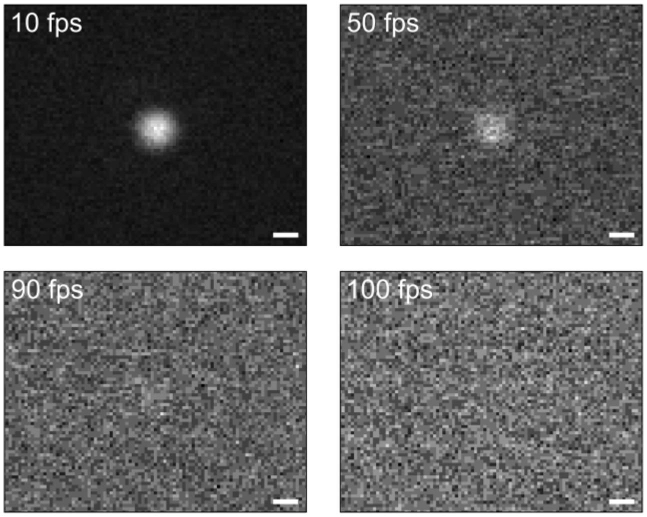


**Supplementary Figure 9** Video-rate imaging of EB-NS with a Si-based camera. An EB-NS was observed with a Si-based camera (Zyla 5.5 sCMOS camera, Oxford Instruments) camera at 10 fps (≈ 100 ms), 50 fps (≈ 20 ms), 90 fps (≈ 11 ms) and 100 fps (≈ 10 ms). The quantum yield of Si-based cameras is high in the visible range, but decreases quickly in the near infrared region for wavelengths larger than 800 nm. While on one hand the particle is still visible at the two slower frame rates, it vanishes as soon as values of 90-100 fps are reached, thus highlighting the benefits of employing an InGaAs camera for NIR imaging. Scale bar = 500 nm.

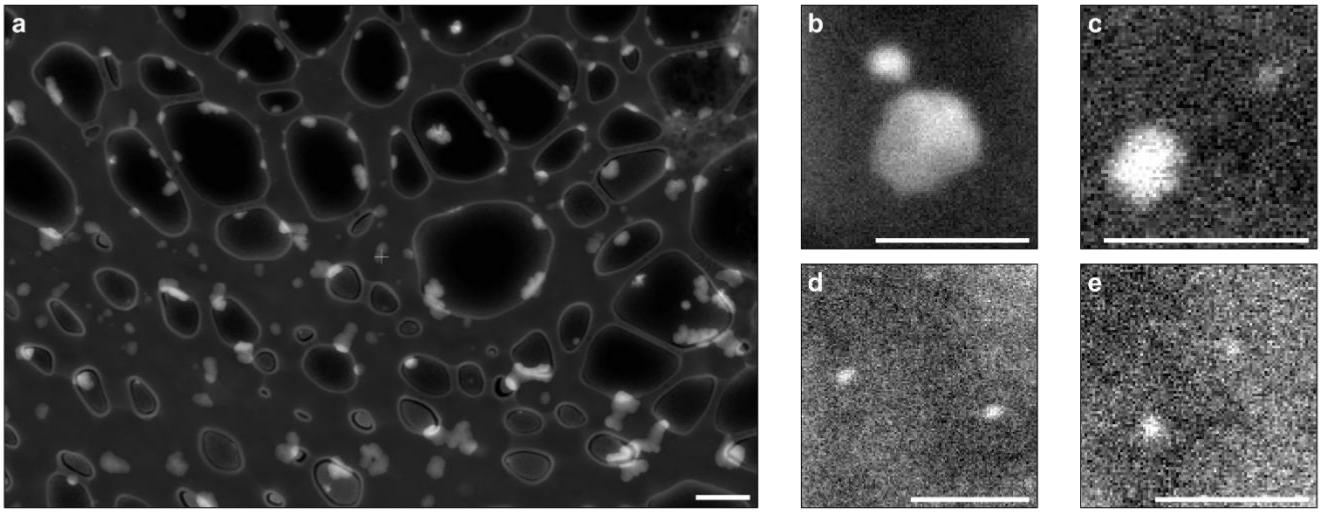


**Supplementary Figure 10** Scanning Transmission Electron Microscopy (STEM) images of EB-NS. **a** Overview image of EB-NS dispersed in isopropanol and spin-coated on a TEM grid. Scale bar = 500 nm. **b-e** Blow-ups of such images of EB-NS. The EB-NS appear to agglomerate during the coating/evaporation process as often seen in TEM images. Scale bar = 100 nm.

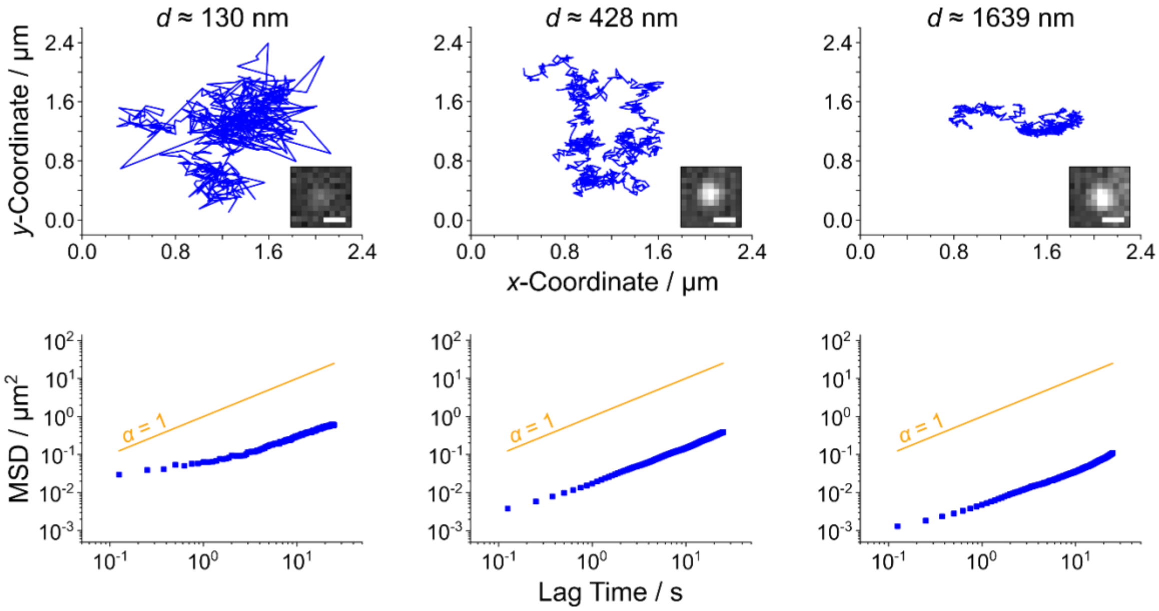


**Supplementary Figure 11** Trajectories and logarithmic MSD curves of three exemplary EB-NS in glycerol. The trajectories and log-log MSDs of three particles of different sizes are shown. In the case of the largest particles, no complete filtration process was performed so that also larger particles and not only nanosheets could be present. Diameters were estimated *via* the protocol used for the size-fluorescence correlation analysis performed in glycerol (Figure 4). As a result, the three particles can be classified as having small, medium (< Abbe limit) and large (> Abbe limit) diameters. The size of the particles assessed (multiple times from different frames) in ImageJ (v. 1.52a) presents diameters of 0.501 µm, 0.551 µm and 0.899 µm (respectively). The Abbe limit of NIR light (900 nm) would be ≈ 450 nm. However, the particles are freely diffusing and therefore most of the time they are not perfectly in focus; the point spread functions should thus be typically bigger/distorted. Scale bar = 1 µm.

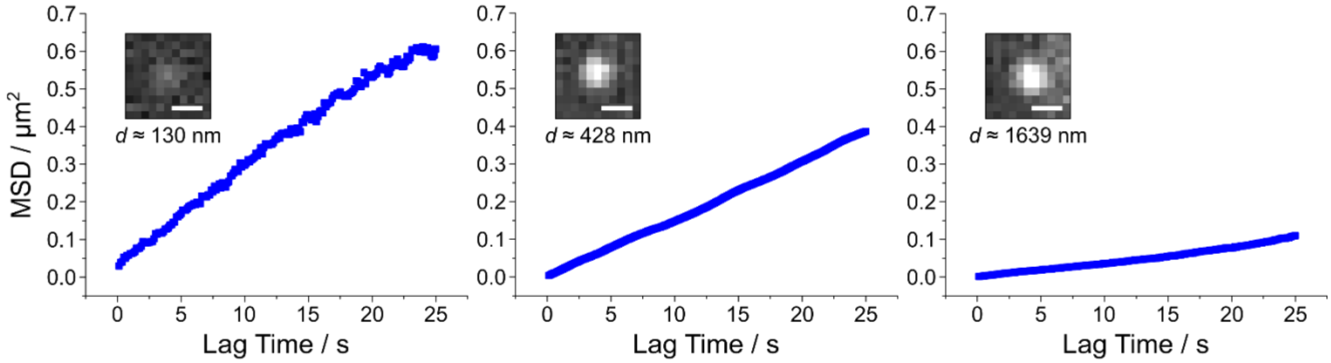


**Supplementary Figure 12** Linear MSD curves of three exemplary EB-NS in glycerol. Linear MSD plots corresponding to the three EB-NS of Supplementary Figure 11 are shown. Scale bar = 1 µm.

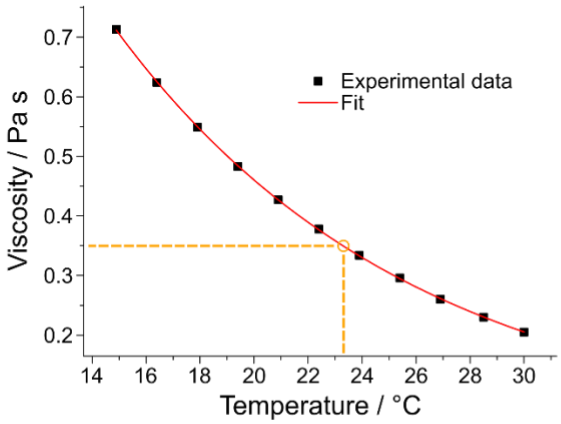


**Supplementary Figure 13** Temperature sweep curve of EB-NS in glycerol. In order to independently assess the viscosity of the glycerol samples charged with EB-NS, a rheometer was used to measure viscosity for different temperatures. An exponential decay fit was chosen to interpolate the raw data ($R_2$ = 0.99994). The sample´s temperature during the NIR imaging experiments was stable and measured ≈ 23.3 °C. The corresponding viscosity value (≈ 0.35 Pa s) was taken from the plot, as indicated by the orange dashed lines.

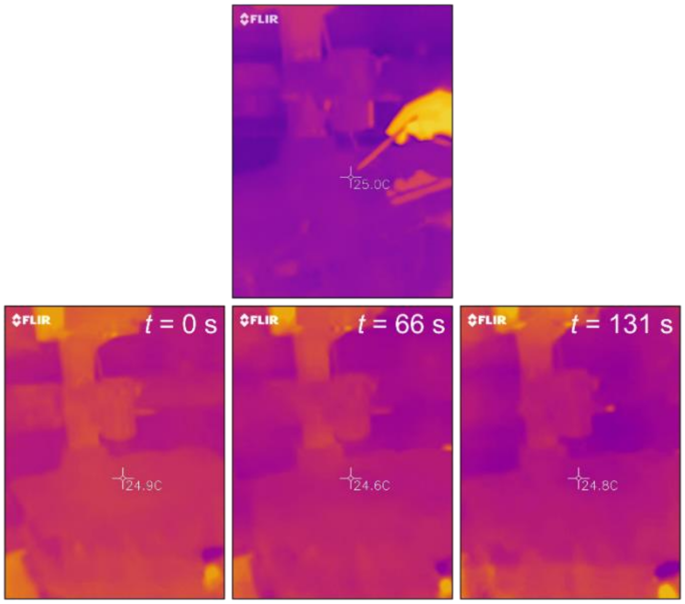


**Supplementary Figure 14** IR monitoring during EB-NS tracking in glycerol. A FLIR camera (FLIR ONE Pro, FLIR Systems, Inc., USA) was employed to observe if EB-NS imaging in glycerol with a 561 nm laser heats the sample. At a laser power of 250 mW, only negligible temperature fluctuations could be observed at $t$ = 0 s (beginning of acquisition), $t$ = 66 s (halfway through the acquisition), and $t$ = 131 s (end of acquisition). The first image shows a hand pointing to the sample holder.

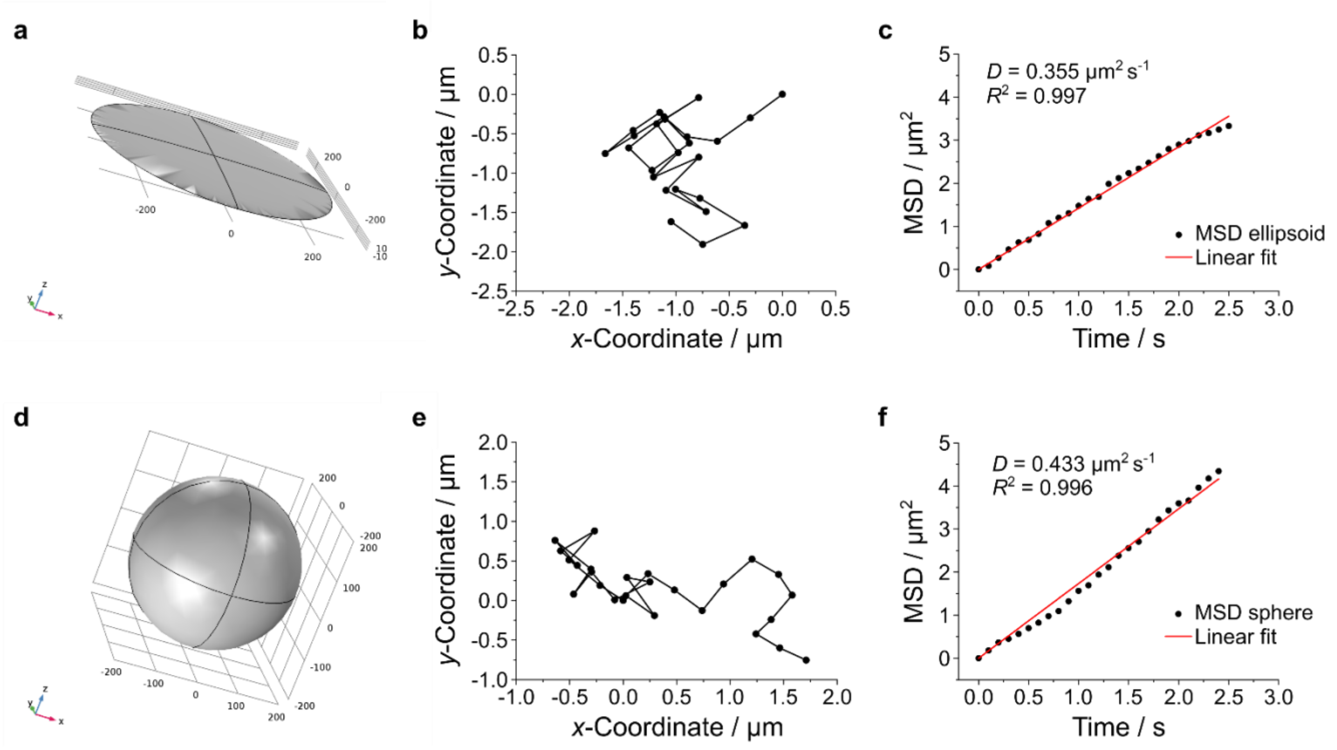


**Supplementary Figure 15** Simulation to assess if the (theoretical) correction factor of the Stokes-Einstein equation for anisotropic particles is correct. **a-c** Spheroid geometry ($d$ = 300 nm), trajectory and MSD of a simulated EB-NS during diffusion in water ($T$ = 300 K). **d-f** Geometry, trajectory and MSD of a spherical particle during diffusion within the same environmental conditions. The radius of this spherical particle was set equivalent to the length of the maximum axis of the EB-NS spheroid, multiplied by the reciprocal of the correction factor ($A$) described in the Supplementary Methods (*i.e.* $1/A \approx 0.67$). This correction factor was derived from theoretical considerations. The MSDs yield diffusion constants (see values in c,f) which are comparable, thus confirming that the used correction factor is reasonable. Simulations were performed in COMSOL (see Supplementary Methods).

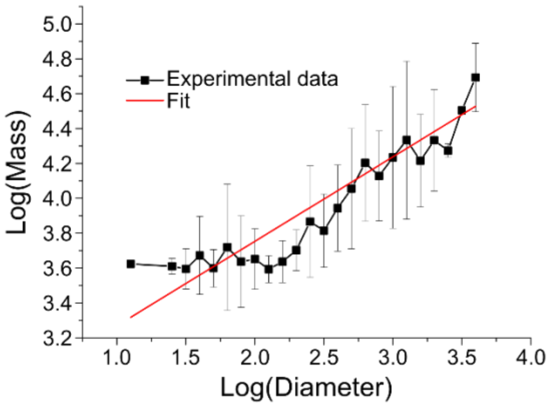


**Supplementary Figure 16** Brightness *vs.* size of EB-NS tracked in glycerol. Mass, as defined by the Trackpy Python package[5,6] (v0.4.2 on Python v3.7.3[7]), is the total integrated brightness of the tracked blob (*i.e.* NIR fluorescence of an EB particle). For these plots, the maximum value along each trajectory was taken to account for the possibility of polarization-dependent excitation/emission. On the *x* axis, the hydrodynamic diameter obtained *via* the Stokes-Einstein equation (with anisotropy correction factor) is displayed. This plot is based on the same data as in Figure 4, but mean fluorescence (mass) values are taken from all data points in 0.1 steps (*x*-axis) to account for the potential spread in height of sheets of similar diameter. As the diameter dominates the diffusion process, this approach allows one to get a better idea if there is a scaling law. The powerlaw fit yields an exponent $\alpha \approx 0.5$ ($R^2$ = 0.77396), which would indicate that fluorescence of EB-NS scales with the square root of particle size. The reported plot is shown as mean ± standard deviation with a step on the *x*-axis of 0.1. *n* = 292 tracked EB-NS.

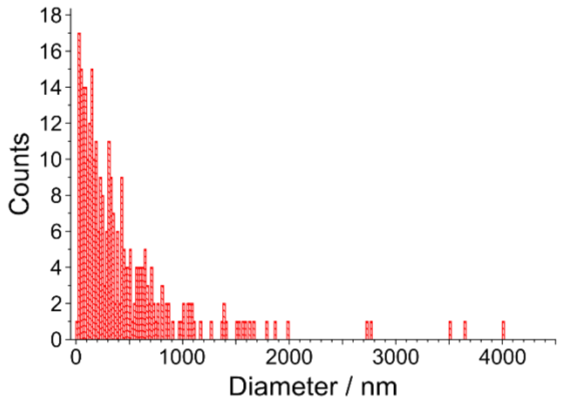


**Supplementary Figure 17** Size distribution of EB-NS in glycerol. The plotted histogram presents hydrodynamic diameter values, which were obtained by size-fluorescence correlation analysis performed in glycerol (*i.e.* Stokes-Einstein with implementation of the correction factor, see Supplementary Methods). Bin size = 20 nm, *n* = 292 tracked EB-NS.

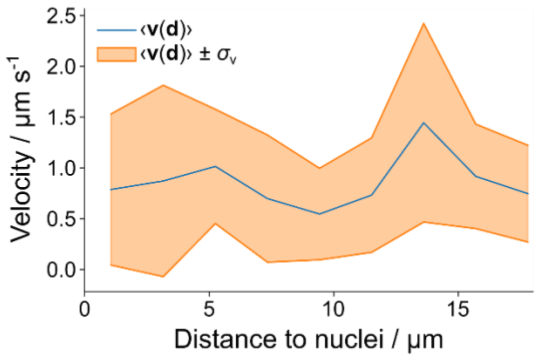


**Supplementary Figure 18** Velocity of EB-NS motion *vs.* distance to closest nucleus in *Drosophila melanogaster* embryos. The mean instantaneous velocity and corresponding variance of EB-NS injected into *Drosophila* embryos is shown as a function of the distance to the closest nuclei encountered during particle motion. The perimeter of nuclei (observable in the GFP channel) was manually measured in ImageJ. The instantaneous velocity (lag time = 0.1 s) and the distance of every point along the trajectory to the closest nucleus were assessed *via* a custom-written Python script. The data set shows that one can use EB-NS to probe local velocities, but every EB-NS might have a unique mechanical environment and therefore there is not a clear trend. Bins width ≈ 2.09 µm, *n* = 38 tracked EB-NS (from 21 different embryos). Tracks for which the nuclei borders could not be clearly depicted were not taken into account.

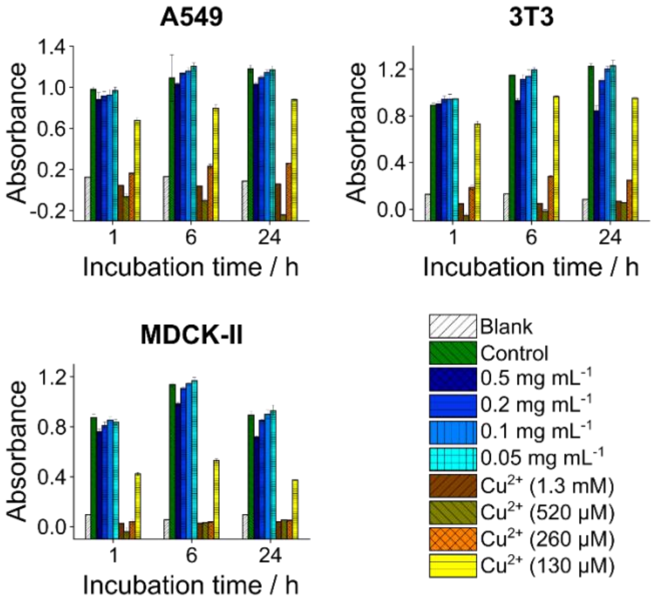


**Supplementary Figure 19** Cell viability in the presence of EB-NS. Cytotoxicity of EB-NS was assessed for 3 different common cell lines (A549, NIH 3T3 and MDCK-II) using a standard assay (see Supplementary Methods). EB-NS did not show significant effects on cell viability, which indicates a high biocompatibility. As a control we used $Cu^{2+}$ ions ($CuSO_4$), which drastically decreased viability. *N* = 4 independent samples (quadruplicates) for each data point, error bars correspond to standard deviation.

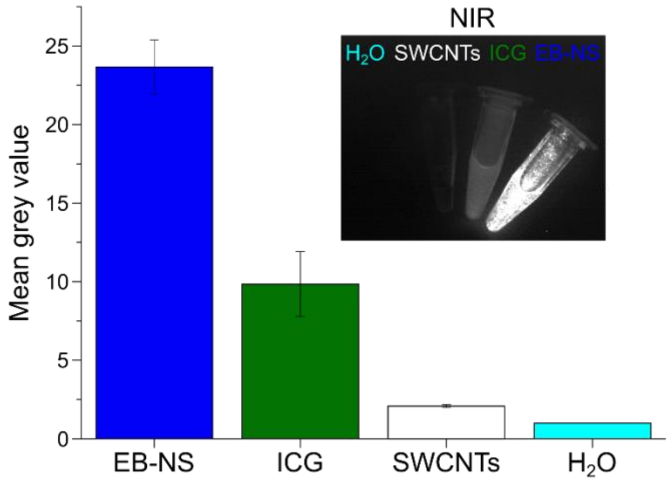


**Supplementary Figure 20** Relative fluorescence intensity of different NIR fluorophores in the low-cost stand-off detection setup. Average total emission intensities (*i.e.* mean grey pixel values) of three different NIR fluorophores in Eppendorf tubes at similar concentration (≈ 0.1 mg mL-1). Data were normalized to the water control. EB-NS, ICG and SWCNTs respectively yielded mean grey values of 23.7 ± 1.7, 9.8 ± 2.1 and 2.1 ± 0.1 (mean ± standard deviation). $N$ = 3 independent samples.

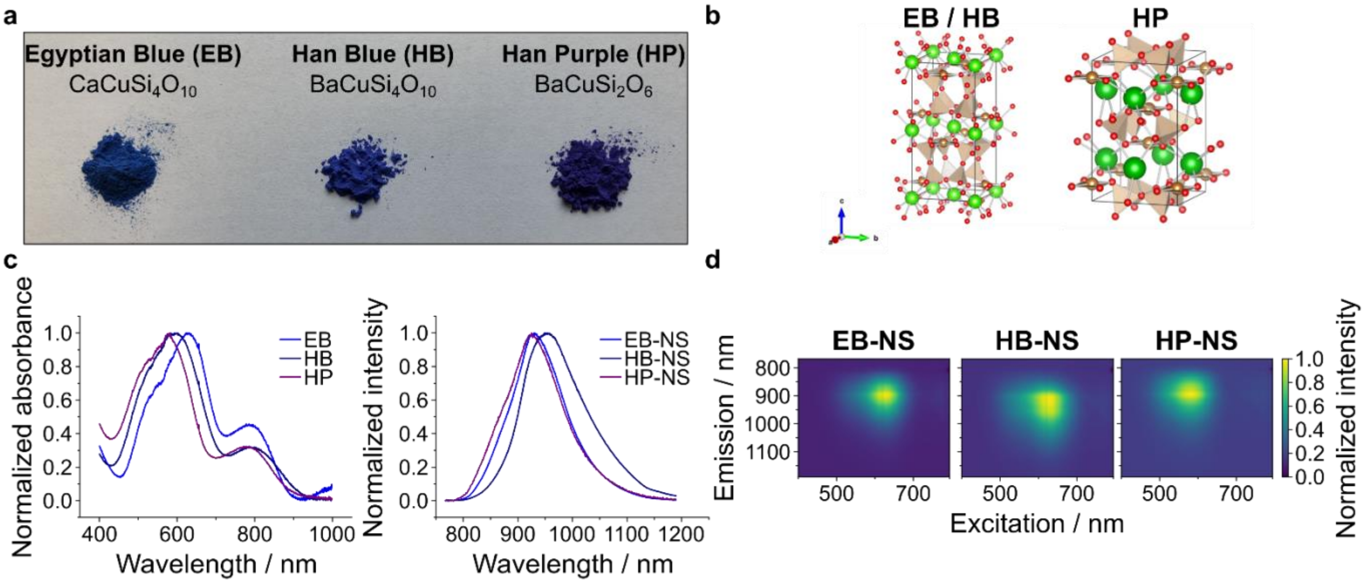


**Supplementary Figure 21** Egyptian Blue (EB), Han Blue (HB) and Han Purple (HP): a family of NIR fluorophores. **a** Picture of bulk, pristine powders of EB, HB and HP. **b** Crystal structures (designed with Vesta (v. 3.4.4)2) of the three pigments: Ca (Ba for HB and HP) is depicted as green, Cu as bronze, O as red spheres, whereas Si is represented in its typical tetrahedral geometry. Colors were chosen according to the CPK color code (Jmol8). **c** Normalized absorption (reflection) of bulk EB, HB and HP powders, and 1D fluorescence spectra of EB, HB and HP nanosheets. **d** Normalized 2D excitation-emission spectra of EB-NS, HB-NS and HP-NS.

**Supplementary Table 1** Fluorescence saturation measurements: saturation power, saturation luminescence intensity and number of luminescence centers per measured particle. Note that the photon numbers were corrected by the efficiency of the optical system *etc.* (see Supplementary Methods).

| Particle number | Saturation excitation power ($P_{sat}$, μW) | Saturation luminescence intensity ($I_{sat}$, × $10^3$ photons $s^{-1}$) | Number of luminescence centers per particle |
|---|---|---|---|
| 1 | 261 | 1250 | 1250 |
| 2 | 387 | 1135 | 1135 |
| 3 | 239 | 1125 | 1125 |
| 4 | 315 | 1008 | 1008 |
| 5 | 294 | 816 | 816 |
| 6 | 421 | 514 | 514 |
| 7 | 408 | 445 | 445 |
| 8 | 347 | 358 | 358 |
| 9 | 431 | 330 | 330 |
| 10 | 381 | 293 | 293 |
| 11 | 219 | 274 | 274 |
| 12 | 359 | 248 | 248 |
| 13 | 418 | 215 | 215 |
| 14 | 312 | 207 | 207 |
| 15 | 403 | 180 | 180 |
| 16 | 386 | 155 | 155 |
| 17 | 209 | 135 | 135 |
| 18 | 287 | 122 | 122 |

## SUPPLEMENTARY METHODS

### Absorption (reflection) spectra of Egyptian Blue (EB), Han Blue (HB) and Han Purple (HP) bulk powders

Absorption spectra were recorded with an AvaSpec-UV/Vis/NIR two channel broad band spectrograph (Avantes) containing a balanced deuterium-halogen lamp (AVALIGHT-DH-S-BAL). Only the signal recorded in the UV/Vis channel is shown, which is equipped with a 2048L UV/Vis spectrometer with a 25 µm slit and a 300 lines $mm^{-1}$ grid. The Avasoft-Full software was used for recording.

### Zeta potential measurements

A Zetasizer Nano S device (Malvern Instruments) was employed for this experiment. The obtained dataset was analysed *via* the Zetasizer software.

### Fluorescence saturation measurements

To estimate the number of luminescent centers in a single EB-NS, we did single particle fluorescence saturation measurements. EB-NS were deposited on the surface of standard glass cover slides. Scanning the surface with a confocal microscope through the diffraction limited focal spot of 1.49 NA objective allowed us to select only the smallest particles with sizes not exceeding the dimensions of the focal spot/resolution limit. Sizes of larger particles clearly exceeded the dimensions of a diffraction limited focal spot and allowed us to distinguish them from smaller particles. After selecting a particle, we measured its fluorescence intensity at different excitation powers. Excitation of nanoparticles that have relatively long excited state lifetimes of the order of 100 µs (Supplementary Figure 7) with a laser that has high repetition rate (20 MHz) allows one to extrapolate saturation of nanoparticle luminescence. The excitation

wavelength was 640 nm. The fluorescence intensity values were normalized to the detection efficiency of the microscope, taking into account the average collection efficiency of light by the 1.49 NA objective lens (70%, given the random orientation of the emission transition dipoles), transmissivity of all the optical elements involved (~50%) and detection efficiency of the single photon avalanche diode (PDM series, MPD) in the appropriate spectral range (~5%). The experimental data were fitted with a typical fluorescence saturation function[9]:

$$I = I_{\mathrm{sat}} \frac{P_{\mathrm{exc}}}{(P_{\mathrm{exc}} + P_{\mathrm{sat}})}, \quad (1)$$

where $P_{exc}$ is the excitation power, $P_{sat}$ is the saturation power, $I_{sat}$ is the nanoparticle luminescence intensity that can be detected at the saturation excitation power, yields the values of $P_{sat}$ and $I_{sat}$ for every saturation curve measured. Supplementary Table 1 shows the obtained values. The obtained saturation luminescence intensity values allow one to estimate the number of luminescent centers *N* per particle. Taking into account the average excited state lifetime $\tau$ (~100 µs, Supplementary Figure 7) and quantum yield $\Phi$ (~0.1[3]), we calculated the number of emitted photons $I_{sat}$ per second into the number of centers:

$$N = \frac{I_{\mathrm{sat}}}{\Phi \tau^{-1}}. \quad (2)$$

Laser source: Supercontinuum laser SC400-4-20, Fianium

Photodetector: single photon avalanche diode PDM series, MPD

Objective lens: Apo N, 60×/1.49 NA oil immersion, Olympus

**Fluorescence lifetime measurements**

Lifetime measurements (frequency domain) were conducted in a well-plate using a Firesting oxygen meter from Pyroscience (Aachen, Germany) at room temperature. The excitation wavelength was set to 620 nm. An excitation frequency of 4 kHz and an LED intensity of 40% were employed.

**Video-rate imaging of EB-NS with a Si-based camera**

EB-NS (previously milled and tip sonicated in water) were filtered with a 0.2 µm syringe filter (Sartorius AG, Germany). To further enhance the concentration of the smallest nanosheets in the sample, the sample vial was placed in a concentrator (Eppendorf® centrifugal vacuum concentrator, Eppendorf, Germany) for 90 min at 50 °C and finally bath sonicated for 20 min to reduce agglomeration. 10 µL were then imaged at the fluorescence microscopy setup on a #1 glass slide with the following settings: 100x objective, 500 mW of 561 nm laser, exposure times and resulting frame rates as specified in the caption of Supplementary Figure 9.

**Scanning Transmission Electron Microscopy (STEM) on EB-NS**

EB-NS (previously milled and tip sonicated in water) were filtered with a 0.45 µm syringe filter (Sartorius AG, Germany) to remove the largest particles from the sample. 10 µL of the so-prepared dispersion were then drop-casted on a TEM grid (ultrathin C film supported by a Lacey carbon film on a 400 mesh Cu grid, Ted Pella Inc, USA) and imaged under a Quattro S (Thermo Fischer Scientific, USA) device with the following settings: working distance = 11.2 mm, horizontal field width = 2-7 µm, 30.00 kV, STEM3+ detector, 8-9×$10^{-6}$ mbar.

**Correlative size-intensity measurements in glycerol**

EB-NS (previously milled and exfoliated/tip sonicated in water) were size-selected by means of two steps of liquid cascade centrifugation[10,11]: in this way we could get rid of unexfoliated EB and increase the monodispersity of the pellet obtained after the last step ($1^{st}$ centrifugation = 240g for 2 h, $2^{nd}$ centrifugation = 2660g for 2 h). 0.5 mL of glycerol (Alfa Aesar, 99+%) were then added to the dried final pellet (≈ 1 mg), and the sample was finally tip sonicated in an ice bath for 2 min at 60% to achieve a homogeneous redispersion. For imaging, 10-20 µL of the glycerol sample were introduced into a flow chamber (µ-Slide VI 0.5 Glass

Bottom, Ibidi, Germany) and placed under our NIR imaging setup. A 100x objective was employed to observe EB-NS at 8 fps (≈ 50 ms exposure time, 1000 frames) with a laser power of 250 mW, which, as shown in Supplementary Figure 14, was not overheating the sample significantly during acquisition. Particle tracking and MSD calculations were performed using a self-written Python script based on the Trackpy package[5,6] (v0.4.2 on Python v3.7.3[7]), whereas the final steps of data analysis were performed on Origin Pro 8.1 software. For identification and linking of particles into trajectories, the following parameters in Trackpy were implemented: memory = 300, minimum number of points (*i.e.* trajectory length) = 300, search_range (*i.e.* maximum displacement between consecutive frames) = 9, diameter (of the tracked blob) = 11. The tracking of the Brownian motion of an EB-NS yields the trajectory of the particle as a set of time-dependent *x* and *y* positions for *N* time steps of length *τ*. From the *x* and *y* positions, the square displacement for the *n*-th time step $\mathbf{r}_n^2$ is calculated as

$$\mathbf{r}_n^2 = \Big(\mathbf{x}(n\cdot\tau) - \mathbf{x}\big((n-1)\cdot\tau\big)\Big)^2 + \Big(\mathbf{y}(n\cdot\tau) - \mathbf{y}\big((n-1)\cdot\tau\big)\Big)^2, \tag{3}$$

where *n* ranges from 1 to *N*. The mean square displacement $\langle\mathbf{r}^2\rangle$ (MSD) is simply the mean over all single time step values:

$$\langle\mathbf{r}^2\rangle = \frac{1}{N}\sum_{n=1}^{N}\mathbf{r}_n^2 \ . \tag{4}$$

Assuming the diffusion is restricted to two dimensions, the diffusion coefficient *D* and the MSD are linked *via* the relation:

$$D = \frac{\langle\mathbf{r}^2\rangle}{4\tau} \ . \tag{5}$$

MSD curves were fitted up to a maximum lag time of 25 s in order to measure the diffusion coefficient of each particle. Finally, from the Stokes-Einstein equation, the Stokes radius *R* is calculated as:

$$R = \frac{k_\mathrm{B}T}{6\pi\eta D} \ , \tag{6}$$

where $\eta$ is the dynamic viscosity of the solvent (Supplementary Figure 13), $T$ the temperature (Supplementary Figure 14) and $k_B$ the Boltzmann constant. The Stokes-Einstein equation is only strictly valid for spherical particles. Due to the high anisotropy of EB-NS, one could assume that Brownian motion is dominated by the diameter of the nanosheets and not the much smaller height. However, all hydrodynamic radii were corrected to account for anisotropy (see below).

**Bulk viscosity measurements of EB-NS in glycerol**

In order to robustly evaluate the bulk viscosity of the glycerol samples, a stress-controlled rheometer (MCR 501, Anton Paar, Austria) that can be operated in oscillatory and rotational mode was used. For a uniform shear rate across the sample, a cone and plate geometry (diameter = 25 mm) was used and the temperature was controlled with a Peltier element. Viscosity of the sample (75 µL) was determined at a constant rotational shear stress of $d\gamma/dt$ = 50 $s^{-1}$ and the temperature was changed from 15 °C to 30 °C at an increment of 1 °C. To ensure reproducibility, the temperature ramp was applied three times starting at 15 °C going to 30 °C, then the reverse order, and finally again from 15 °C to 30 °C.

**Estimation of the hydrodynamic radius of EB-NS**

To account for the (non-spherical) asymmetry of EB-NS, a correction factor $A$ was applied to the general Stokes-Einstein equation:

$$D = \frac{k_B T}{6\pi\mu r} = A\frac{k_B T}{6\pi\mu h}, \quad (7)$$

where $h$ is the length of the main axis of the particle and $r$ is the hydrodynamic radius of a sphere of equivalent diffusivity. To estimate the correction factor, the theoretical framework of Happel *et al.* was used[12]. In short, the geometry of a given EB-NS was approximated with the shape of a nanometer-sized spheroid containing two symmetric long axis $h$ (particle width) as well as a short axis $a$ (particle height). By calculating the resistance of such a particle within a

fluid of unidirectional flow parallel or perpendicular to the particle's symmetry axis, one is able to derive an expression for the hydrodynamic radius $R$ of a sphere with equivalent diffusivity by:

$$R_{-} = \frac{8h}{3}\frac{1}{\left[\frac{2\varphi}{1-\varphi^2}+\frac{2(1-2\varphi^2)}{(1-\varphi^2)^{3/2}}\tan^{-1}\left(\frac{\sqrt{1-\varphi^2}}{\varphi}\right)\right]} \text{ (parallel to symmetry axis)} \quad (8)$$

and

$$R_{\perp} = \frac{8h}{3}\frac{1}{\left[-\frac{\varphi}{1-\varphi^2}-\frac{2\varphi^2-3}{(1-\varphi^2)^{3/2}}\sin^{-1}\left(\sqrt{1-\varphi^2}\right)\right]} \text{ (perpendicular to symmetry axis) , (9)}$$

with $\varphi$ delineating the axis ratio $\varphi = a/h$. For $\varphi$ we utilized the height/width values gained from our AFM-measurements for EB-NS (see Figure 1). A linear fit yields $\varphi$ = 0.035 or $R_{-}$ = 0.85$h$ and $R_{\perp}$ = 0.58$h$, respectively. Finally, the factor $A$ could be calculated by taking the mean of all axis values:

$$R_{\text{mean}} = \frac{R_{-} + 2R_{\perp}}{3} = \frac{0.85 + 2\cdot 0.58}{3}h = \frac{1}{A}h \;\rightarrow A = 1.49 \quad (10)$$

The actual hydrodynamic diameters of the measured EB-NS are therefore larger than expected from the pure Stokes-Einstein equation.

**3D simulation of EB particle diffusion**

Simulations of diffusive EB particles were performed with COMSOL (v 5.5). For this purpose, the Fluid-Structure-Interaction (*fsi*) package was used to create a 500 x 500 x 500 µm$^3$ sized, cubic water box ($T$ = 300 K, $\mu$ = 0.001 Pa s, $\rho$ = 997 kg m$^{-3}$, free wall boundary conditions) in which

*e.g.* a spheroidic "EB" particle ($h$ = 300 nm, $a$ = 10.44 nm, $\rho$ = 8960 kg m$^{-3}$) was placed in its center to mimic Brownian motion. Consequently, we implemented stochastic forces applied from the fluid on the particle's surface ($\mathbf{F}_{\text{mean}}$ ≈ 1 N m$^{-2}$, approximated from test simulations to match the Stokes-Einstein relation) and started the simulation process by setting the solver step size (frame rate) to Δ$t$ = 0.1 s. The motion of the particle was then tracked by analyzing its

center of mass in each step size and the traces were used to calculate the MSD and the particle's respective diffusion coefficient. This process was compared to particles of different geometry *e.g.* for $r = 300/A = 201$ nm spheres (corresponding to the correction factor expected from the theory above).

**Cytotoxicity tests of EB-NS**

A549, NIH 3T3 and MDCK II cell lines were employed in this study.

- A549 cell line and NIH 3T3 cell line: D10F+ (DMEM +4.5 g $L^{-1}$ glucose with L-glutamin (4 mM), 10% fetal bovine serum and 100 µg $mL^{-1}$ Pen-Strep), trypsin/EDTA (0.05/0.05%) solution;
- MDCK II cell line: M10F+ (EMEM with Earle's salts with L-glutamin (2 mM), 10% fetal bovine serum and 100 µg $mL^{-1}$ Pen-Strep), trypsin/EDTA (0.25/0.05%) solution.

The cell viability (MTS) assay was performed using CellTiter 96® AQueous One Solution Cell Proliferation Assay (Promega, G3580). 2 mL (≈ 4 mg) of a 6 h-tip sonicated sample of EB-NS in water (2 mg $mL^{-1}$) was centrifuged (10 min, 13100 RCF). The supernatant was removed and the so-obtained pellet was re-dispersed in 400 µL, in order to reach a concentration of ≈ 10 mg $mL^{-1}$ of EB-NS. The sample was diluted to 5% (0.5 mg $mL^{-1}$), 2% (0.2 mg $mL^{-1}$), 1% (0.1 mg $mL^{-1}$) and 0.5% (0.05 mg $mL^{-1}$).

A 13 mg $mL^{-1}$ (*i.e.* 52 mM) water solution of $CuSO_4 \cdot 5H_2O$ (98%, J&K Scientific) was used as a reference to assess the maximum sensitivity of the cells to $Cu^{2+}$ ions. 0.2% (100 µM) corresponds to the copper amount in 2% of the 2 mg $mL^{-1}$ EB-NS dispersion (*i.e.* 0.04 mg $mL^{-1}$ of EB-NS). Therefore 2.5% (1.3 mM), 1% (520 µM), 0.5% (260 µM) and 0.25% (130 µM) correspond to the EB-NS concentrations of 0.5 mg $mL^{-1}$, 0.2 mg $mL^{-1}$, 0.1 mg $mL^{-1}$, 0.05 mg $mL^{-1}$, respectively. The $Cu^{2+}$ concentrations for this experiment have been shown to cause varying degrees of cytotoxicity in literature[13]. 2% (v/v) deionized water

was employed as positive control. Cells were initially incubated in a 96-well plate at $1.2\times10^3$ cells per well for 24 h in M10F$^-$ Media at 37 °C and 7.5% $CO_2$ (MDCK II), or in D10F$^-$ Media at 37 °C and 5% $CO_2$ (A549 and NIH 3T3). A suspension of EB-NS was diluted to the different concentrations in appropriate media and added to the cells to be incubated for 1, 6 or 24 h. Viability of the cell samples was determined by MTS-assay.

**1D and 2D fluorescence spectra of EB, HB and HP**

6 h-tip sonicated EB, HB and HP samples in isopropanol were centrifuged for 2 h at 240g to remove unexfoliated bulk material. 200 µL of each supernatant were then placed in a 96-well-plate and the excitation wavelength of the monochromator was set at 615 nm for all samples. For the acquisition of 2D spectra, the following parameters were employed: excitation wavelength range = 400-800 nm, steps = 5 nm, integration time = 3 s. 2D Spectra were normalized to the maximum intensity and corrected for the quantum efficiency of the detector as well as the spectral irradiance of the xenon lamp of the monochromator using a self-written Python script.

## SUPPLEMENTARY REFERENCES


1. Chakoumakos, B. C., Fernandez-Baca, J. A. & Boatner, L. A. Refinement of the Structures of the Layer Silicates. *J. Solid State Chem.* **103**, 105–113 (1993).

2. Momma, K. & Izumi, F. VESTA 3 for three-dimensional visualization of crystal, volumetric and morphology data. *J. Appl. Crystallogr.* **44**, 1272–1276 (2011).

3. Accorsi, G. *et al.* The exceptional near-infrared luminescence properties of cuprorivaite (Egyptian blue). *Chem. Commun.* 3392–3394 (2009).

4. Pabst, A. Structures of some tetragonal sheet silicates. *Acta Crystallogr.* **12**, 733–739 (1959).

5. Crocker, J. C. & Grier, D. G. Methods of digital video microscopy for colloidal studies. *J. Colloid Interface Sci.* **179**, 298–310 (1996).

6. Allan, D. *et al.* soft-matter/trackpy: Trackpy v0.4.2. (2019) doi:10.5281/ZENODO.3492186.

7. Welcome to Python.org. https://www.python.org/.

8. Colors. http://jmol.sourceforge.net/jscolors/.

9. Plakhotnik, T. & Gruber, D. Luminescence of nitrogen-vacancy centers in nanodiamonds at temperatures between 300 and 700 K: Perspectives on nanothermometry. *Phys. Chem. Chem. Phys.* **12**, 9751–9756 (2010).

10. Backes, C. *et al.* Production of highly monolayer enriched dispersions of liquid-exfoliated nanosheets by liquid cascade centrifugation. *ACS Nano* **10**, 1589–1601 (2016).

11. Backes, C. *et al.* Guidelines for Exfoliation, Characterization and Processing of Layered Materials Produced by Liquid Exfoliation. (2017) doi:10.1021/acs.chemmater.6b03335.

12. Happel, J. & Brenner, H. The Motion of a Rigid Particle of Arbitrary Shape in an Unbounded Fluid. 159–234 (1983) doi:10.1007/978-94-009-8352-6_5.

13. Tchounwou, P. B., Newsome, C., Williams, J. & Glass, K. Copper-Induced Cytotoxicity and Transcriptional Activation of Met Ions Biol Med . 2008 ; 10: 285–290. Stress Genes in Human Liver Carcinoma (HepG2 ) Cells. *Met. Ions Biol. Med.* **10**, 285–290 (2008).
