# Supplementary material for: Exfoliated near infrared fluorescent silicate nanosheets for (bio)photonics
Source: Nat Commun. 2020 Mar 20;11:1495. doi: 10.1038/s41467-020-15299-5 (PMC7083911; doi:10.1038/s41467-020-15299-5)
Supplement: Supplementary file 3 — Reporting Summary [file 41467_2020_15299_MOESM3_ESM.pdf]

nature research

Corresponding author(s): Sebastian Kruss

Last updated by author(s): Feb 17, 2020

Reporting Summary

Nature Research wishes to improve the reproducibility of the work that we publish. This form provides structure for consistency and transparency in reporting. For further information on Nature Research policies, see Authors & Referees and the Editorial Policy Checklist .

Statistics

For all statistical analyses, confirm that the following items are present in the figure legend, table legend, main text, or Methods section.

n/a	Confirmed	
☐	☒	The exact sample size (*n*) for each experimental group/condition, given as a discrete number and unit of measurement
☐	☒	A statement on whether measurements were taken from distinct samples or whether the same sample was measured repeatedly
☒	☐	The statistical test(s) used AND whether they are one- or two-sided<br>*Only common tests should be described solely by name; describe more complex techniques in the Methods section.*
☒	☐	A description of all covariates tested
☐	☒	A description of any assumptions or corrections, such as tests of normality and adjustment for multiple comparisons
☐	☒	A full description of the statistical parameters including central tendency (e.g. means) or other basic estimates (e.g. regression coefficient) AND variation (e.g. standard deviation) or associated estimates of uncertainty (e.g. confidence intervals)
☒	☐	For null hypothesis testing, the test statistic (e.g. *F*, *t*, *r*) with confidence intervals, effect sizes, degrees of freedom and *P* value noted<br>*Give P values as exact values whenever suitable.*
☒	☐	For Bayesian analysis, information on the choice of priors and Markov chain Monte Carlo settings
☒	☐	For hierarchical and complex designs, identification of the appropriate level for tests and full reporting of outcomes
☒	☐	Estimates of effect sizes (e.g. Cohen's *d*, Pearson's *r*), indicating how they were calculated

Our web collection on statistics for biologists contains articles on many of the points above.

Software and code

Policy information about availability of computer code

Data collection	AFM --> Asylum Research (v. 15.01.103)<br>Spectrometer --> Andor Solis (v. 4.29.30012.0)<br>Cameras --> Xeneth (v. 2.6.0.1138), Andor Solis (v. 4.29.30012.0)<br>SEM --> Leo 32 (v. 02.03), Thermoscientific Quattro S (v. 15.2.1)<br>Stand-off detection setup --> ThorCam (v. 3.2.0)<br>Modelling of cuprorivaite monolayer structure --> VESTA (v. 3.4.4)<br>Absorption spectrum --> Avasoft-Full (v. 8)<br>Zeta potential measurements --> Zetasizer (v. 7.12)<br>Lifetime measurements --> Pyro Oxygen Logger (v. 3.213)<br>Fluorescence saturation measurements --> custom-written Labview code<br>Correction factor for diffusion experiments in glycerol --> COMSOL (v. 5.5)<br>Laser Diffraction Particle Size Analyzer --> Beckman&Coulter LS 13320 (v. 6.01)
Data analysis	AFM --> Gwyddion (v. 2.51)<br>2D spectra --> self-written Python code (v. 3.7.3)<br>Analysis of spectra, AFM data, toxicity tests, size-fluorescence correlation methods --> OriginPro (v. 8.1)<br>Stand-off detection brightness evaluation, size-fluorescence correlation methods & nIR imaging --> ImageJ 1.52i<br>Fluorescence saturation measurements --> custom-written Matlab code<br>Single-particle tracking & MSD analysis --> self-written Python script based on the Trackpy package (v. 0.4.2 on Python v. 3.7.3)

For manuscripts utilizing custom algorithms or software that are central to the research but not yet described in published literature, software must be made available to editors/reviewers. We strongly encourage code deposition in a community repository (e.g. GitHub). See the Nature Research guidelines for submitting code & software for further information.

Data

Policy information about availability of data

All manuscripts must include a data availability statement. This statement should provide the following information, where applicable:

- Accession codes, unique identifiers, or web links for publicly available datasets
- A list of figures that have associated raw data
- A description of any restrictions on data availability

The data that support the findings of this study are available from the corresponding author upon reasonable request

Field-specific reporting

Please select the one below that is the best fit for your research. If you are not sure, read the appropriate sections before making your selection.

[x] Life sciences   [ ] Behavioural & social sciences   [ ] Ecological, evolutionary & environmental sciences

For a reference copy of the document with all sections, see nature.com/documents/nr-reporting-summary-flat.pdf

Life sciences study design

All studies must disclose on these points even when the disclosure is negative.

Sample size	Cell studies: n=4 independent samples for each datapoint<br>Drosophila embryo studies: 3 independent sessions of injections on multiple embryos were performed<br>Arabidopsis thaliana studies: around 20 leaves (3 independent plants) have been infiltrated
Data exclusions	Cell and Arabidopsis thaliana studies: no data was excluded<br>Drosophila embryos: data was exluded when the position of the imaged nuclei (z-position in wide-field setup sometimes not clear) could not be unequivocally identified at the microscope setup
Replication	Cell studies: reproducibility was confirmed by the repetition of the same cell experiments<br>Drosophila embryo studies: on each of the 3 sessions of injections, between 9 and 11 embryos were injected. Limited by injection efficiency and nucleus staining quality 38 trajectories out of 21 embryos could be followed<br>Arabidopsis thaliana studies: the protocol followed for the infiltration procedure had to be repeated several times due to its non-trivial execution
Randomization	Cell and Arabidopsis thaliana studies: no randomization was necessary<br>Not used in Drosophila embryo studies, as well
Blinding	For cell, Drosophila embryo and Arabidopsis thaliana studies, blinding was not relevant

Reporting for specific materials, systems and methods

We require information from authors about some types of materials, experimental systems and methods used in many studies. Here, indicate whether each material, system or method listed is relevant to your study. If you are not sure if a list item applies to your research, read the appropriate section before selecting a response.

Materials & experimental systems

n/a	Involved in the study	
x		Antibodies
	x	Eukaryotic cell lines
x		Palaeontology
	x	Animals and other organisms
x		Human research participants
x		Clinical data

Methods

n/a	Involved in the study	
x		ChIP-seq
x		Flow cytometry
x		MRI-based neuroimaging

Eukaryotic cell lines

Policy information about cell lines

Cell line source(s)	A549 --> ECACC 86012804 ; MDCK-II --> ECACC 00062107 ; NIH-3T3 --> DSMZ ACC 59
Authentication	Cells were ordered directly from the cell banks, therefore no authentication was performed
Mycoplasma contamination	Cell lines regularly test negative for mycoplasma contamination
Commonly misidentified lines (See ICLAC register)	None

Animals and other organisms

Policy information about studies involving animals; ARRIVE guidelines recommended for reporting animal research

Laboratory animals	Drosophila melanogaster
Wild animals	This study did not involve wild animals
Field-collected samples	This study did not involve samples collected from field
Ethics oversight	No ethical approval was required. This study did not involve animals which need ethical approval

Note that full information on the approval of the study protocol must also be provided in the manuscript.
