# Supplementary material for: Exfoliated near infrared fluorescent silicate nanosheets for (bio)photonics
Source: Nat Commun. 2020 Mar 20;11:1495. doi: 10.1038/s41467-020-15299-5 (PMC7083911; doi:10.1038/s41467-020-15299-5)
Supplement: Supplementary file 2 — Peer Review File [file 41467_2020_15299_MOESM2_ESM.pdf]

Editorial Note: This manuscript has been previously reviewed at another journal that is not operating a transparent peer review scheme. This document only contains reviewer comments and rebuttal letters for versions considered at Nature Communications.

Reviewers' Comments:

Reviewer #3:
Remarks to the Author:
The authors have addressed all my comments therefore I recommend publication to Nature Communications.

Reviewer #4:
Remarks to the Author:
Selvaggio et al present the development and application of nano sheets derived from Egyptian Blue dye as NIR sensor for biomedical application. They carefully characterised the performance of these new nanosheets incl. their size dependence. Since these sheets are free from any bleaching and little sensitive to reducing agents, they are promising materials for bioimaging in the nIR.

The comments by referee #2 are well addressed. The manuscript has been significantly improved and should be published as it is.

--- Typos:
page 7, last line: Please check the number of luminescent centres.
